# Aharonov–Bohm interference in a ${\pmb{\mathbb{Z}}}_{\bf{2}}$ lattice gauge theory on a hybrid qubit–oscillator quantum computer

**DOI:** 10.1038/s41567-026-03400-6

**Published:** 2026-09-25

**Authors:** S. Saner, O. Băzăvan, D. J. Webb, G. Araneda, C. J. Ballance, R. Srinivas, D. M. Lucas, A. Bermúdez

**Affiliations:** 1 Department of Physics, University of Oxford, Oxford, UK; 2 Instituto de Física Teórica, UAM-CSIC, Universidad Autónoma de Madrid, Madrid, Spain

**Keywords:** Quantum simulation, Condensed-matter physics, Quantum information

## Abstract

Lattice gauge theories, which have both dynamical matter and gauge fields, are strongly coupled problems that lie beyond the reach of classical computation. Quantum simulations may provide a tractable approach for studying lattice gauge theories, but implementing gauge-invariant encodings and real-time evolution remains experimentally challenging. Here we demonstrate a resource-efficient encoding of a ${{\mathbb{Z}}}_{2}$ lattice gauge theory using a hybrid qubit–oscillator trapped-ion quantum device, with the qubits representing the gauge fields and the vibrational modes of the ions encoding the bosonic matter fields. We use synthetic dimensions to construct higher dimensional lattice geometries, and we combine digital and analogue techniques to prepare the initial states, realize the gauge-invariant real-time evolution and measure the relevant observables. After observing dynamics obeying Gauss’s law in a ${{\mathbb{Z}}}_{2}$ link, we extend this approach to a loop geometry formed by two qubits and two oscillators. In this quasi-two-dimensional set-up, we observe Aharonov–Bohm interference with dynamical gauge fields encoding the magnetic flux, thereby demonstrating the interplay between charge and flux. Our results establish a path for scalable quantum simulations of lattice gauge theories in higher dimensions.

## Abstract (web summary)

Lattice gauge theories are difficult to study using classical computational methods. A dynamic quantum simulation of a lattice gauge theory on a two-site loop has now been demonstrated using a hybrid qubit–oscillator trapped-ion quantum computer.

## Main

Gauge theories provide a unifying framework for understanding the interactions of matter and gauge fields in nature, ranging from high-energy physics^1^ to condensed matter^2^. Matter fields interact through gauge fields according to laws that are invariant under specific local symmetries. In the Standard Model of particle physics^3^, for example, the electromagnetic, weak and strong forces are mediated by gauge fields in three spatial and one temporal dimension (3 + 1D)^4^. These gauge fields arise from local symmetries described by the gauge groups U(1), SU(2) and SU(3), respectively. Although gauge theories underpin particle physics, they also arise as effective descriptions of strongly correlated condensed matter systems. However, solving them in physically relevant regimes is often challenging. In particle physics, phenomena like deep inelastic scattering and dense nuclear matter, which involve real-time dynamics and finite-density, respectively, elude standard Monte Carlo approaches^5,6^. Similarly, in condensed matter, strongly correlated systems and topological order require non-perturbative approaches.

Lattice gauge theories (LGTs), which discretize models onto lattices, with the gauge fields on links and the matter at vertices, provide a powerful framework for addressing these challenges^7–9^. Among LGTs, those with discrete ${{\mathbb{Z}}}_{2}$ groups^10,11^ are particularly important in condensed matter physics, as they describe frustrated antiferromagnets^12^, high-temperature superconductors^13,14^ and quantum spin-liquid phases^15^. In the context of quantum information processing, ${{\mathbb{Z}}}_{2}$ LGTs^10,11^ are relevant to topological quantum error correction^16,17^.

The richness of phenomena associated with ${{\mathbb{Z}}}_{2}$ LGTs grows with the dimensionality of the system. For 1 + 1D, we can observe gauge-invariant tunnelling along with signatures of confinement, where the propagation of matter fields starts to be restricted^18,19^. In 2 + 1D dimensions, the lattice can be pierced by magnetic flux, resulting in quasiparticle excitations called visons^14^. In these electrically and magnetically dominated phases, the real-time dynamics of the gauge fields are intertwined with confined or deconfined matter fields. In 3 + 1D, the study of flux sources, which are discrete analogues of magnetic monopoles, becomes possible. Understanding these strongly correlated, real-time dynamics is a classically intractable challenge, and the use of quantum simulators could offer a solution^20–27^.

In recent years, experimental demonstrations of quantum simulations of real-time dynamics in ${{\mathbb{Z}}}_{2}$ LGTs have mainly focused on 1 + 1D geometries, where visons and topological effects are absent. These experiments have implemented analogue Floquet schemes with cold atoms in a Hubbard double-well link^28^ or digital gate-based schemes with superconducting qubits using linear connectivity^29,30^. Both analogue and digital approaches have also used effective formulations of 2 + 1D systems by enforcing Gauss’s law to eliminate matter degrees of freedom^31–34^. These approaches, although offering insights, cannot be used to experimentally observe the interplay between dynamical gauge fields and matter fields, which gives rise to the attachment of electric field lines to charges via Gauss’s law and to flux–charge interactions in higher dimensional systems.

**Fig. 1:**
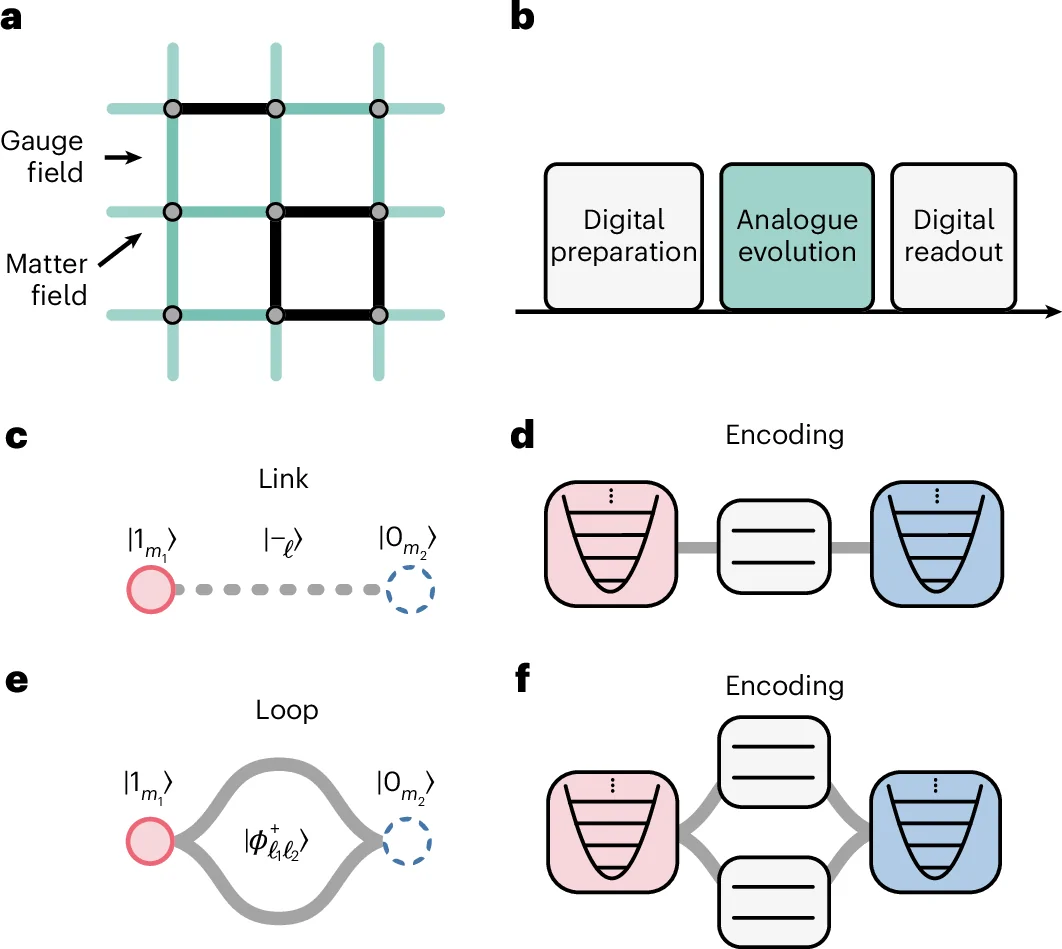
Quantum simulation of a ${{\mathbb{Z}}}_{2}$ LGT using the hybrid encoding scheme. **a**, The gauge fields reside on the links connecting the matter sites, which are at the nodes. The fundamental building blocks—the gauge–matter link and the loop—are highlighted in black. **b**, The quantum simulation pulse sequence consists of a digital preparation of the hybrid system used to encode the LGT followed by analogue evolution under the gauge-invariant Hamiltonian ${\hat{H}}_{{{\mathbb{Z}}}_{2}}$ and concludes with digital read-out. **c**, Illustration of a single gauge–matter link in state $\left\vert {1}_{{m}_{1}},{-}_{\ell },{0}_{{m}_{2}}\right\rangle$. **d**, The corresponding encoding into the hybrid qubit–oscillator system. The matter sites are represented by two motional modes of a harmonically confined trapped ion. The gauge field is encoded in two levels of its internal electronic structure. **e**, Illustration of a ${{\mathbb{Z}}}_{2}$ loop in state $\vert {1}_{{m}_{1}},{\Phi }_{{\ell }_{1}{\ell }_{2}}^{+},{0}_{{m}_{2}}\rangle$ with the gauge fields prepared in the entangled state $\vert {\Phi }_{{\ell }_{1}{\ell }_{2}}^{+}\rangle$. **f**, Corresponding extension of the hybrid encoding with another gauge field.

Here, to address these gaps, we demonstrate a quantum simulation of a ${{\mathbb{Z}}}_{2}$ LGT using a trapped-ion processor with a hybrid qubit–oscillator architecture, where qubits encode gauge fields via electronic levels and oscillators represent matter fields via vibrational modes^35–38^. Our approach is hybrid in two ways. First, it combines discrete and continuous variables to efficiently simulate the target LGT. Complementary recent work by the Ion Trap Group at the University of Maryland likewise uses a qubit–oscillator architecture to simulate the Yukawa model^39^. More broadly, qubit–qudit hybrid approaches have been explored. These approaches enable LGT studies of truncated U(1) models and real-time dynamics via Trotterized evolutions^40^. Second, our method integrates digital operations (single-qubit, two-qubit and qubit–oscillator gates) for initialization, error suppression and measurement with analogue evolution under an engineered ${{\mathbb{Z}}}_{2}$-invariant Hamiltonian (Fig. 1a,b).

The hybrid architecture enables the realization of lattice gauge geometries by introducing synthetic spatial dimensions via qubit–oscillator couplings. We demonstrate correlated real-time dynamics of a matter charge and an attached electric field line, consistent with Gauss’s law in a minimal 1 + 1D ${{\mathbb{Z}}}_{2}$ link encoded in a single trapped ion. Extending this approach to a two-ion crystal, we added a second ${{\mathbb{Z}}}_{2}$ link to form a loop, thereby enabling fundamental 2 + 1D gauge dynamics. In this configuration, we prepared entangled gauge fields representing a gauge-invariant ${{\mathbb{Z}}}_{2}$ flux piercing the loop and demonstrate that charge dynamics is inhibited by destructive interference depending on the flux state (the presence of a vison); this provides a direct observation of the ${{\mathbb{Z}}}_{2}$ Aharonov–Bohm effect.

## LGT encoding

We take a bottom-up approach to LGTs, starting with the simplest element: a ${{\mathbb{Z}}}_{2}$ link, such that two matter sites are coupled via a gauge field (Fig. 1c). From this basic unit, we progressively build more complex lattice geometries link by link. Central to our approach is the encoding of the ${{\mathbb{Z}}}_{2}$ link within a qubit–oscillator quantum system (Fig. 1d). Here the gauge field *ℓ* is represented by a qubit, which has two states $\left\vert {\uparrow }_{\ell }\right\rangle$ and $\left\vert {\downarrow }_{\ell }\right\rangle$ acted on by Pauli matrices ${\hat{\sigma }}_{\ell }^{x,y,z}$. The matter sites *m*_*i*_ with *i* ∈ {1, 2}, are encoded in harmonic oscillators, which have bosonic particle creation (${\hat{a}}_{{m}_{i}}^{\dagger }$) and annihilation (${\hat{a}}_{{m}_{i}}$) operators. This encoding enables the engineering of synthetic dimensions while naturally incorporating bosonic matter to support several excitations at each site, thereby extending the proposal in ref. ^35^.

We consider an LGT that satisfies a local ${{\mathbb{Z}}}_{2}$ gauge symmetry, which in the single-link case is generated by ${\hat{G}}_{{m}_{i}}={\hat{\sigma }}_{\ell }^{x}{\hat{P}}_{{m}_{i}}$ at each site and at all times, where ${\hat{P}}_{{m}_{i}}=\exp \,({{\mathrm{i}}}{\pi}{\hat{a}}_{{m}_{i}}^{\dagger }{\hat{a}}_{{m}_{i}})=1-2{\hat{Q}}_{{m}_{i}}$ is the matter parity. In this representation, ${\hat{\sigma }}_{\ell }^{x}$ provides a convenient basis for the gauge field, with eigenstates $\left\vert {+}_{\ell }\right\rangle$ and $\left\vert {-}_{\ell }\right\rangle$ corresponding to the presence (+) or absence (−) of an electric field line connecting the two matter sites. Similarly, ${\hat{Q}}_{{m}_{i}}$ defines a convenient local basis for the matter (charge) degrees of freedom, with eigenvalues ${q}_{{m}_{i}}\in \{0,1\}$, such that states with an even (odd) number of bosons $\vert 2{k}_{{m}_{i}}\rangle$ ($\vert {(2k+1)}_{{m}_{i}}\rangle$) have charge ${q}_{{m}_{i}}=0$ (1). However, although physical states obey Gauss’s law and must, therefore, be eigenstates of ${\hat{G}}_{{m}_{i}}$, the gauge and matter field can be entangled and need not be eigenstates of ${\hat{\sigma }}_{\ell }^{x}$ or ${\hat{P}}_{{m}_{i}}$ individually.

When a matter particle is charged under a gauge group, it acquires an Aharonov–Bohm-type phase as it moves between lattice sites, which is determined by the line integral of the corresponding gauge field^41^. In the ${{\mathbb{Z}}}_{2}$ case, this leads to a real-time evolution where matter charges and gauge fields are coupled through the Hamiltonian:

1 $${\hat{H}}_{{{\mathbb{Z}}}_{2}}^{{\rm{link}}}=\left(J{\hat{a}}_{{m}_{1}}^{\dagger }{\hat{\sigma }}_{\ell }^{z}{\hat{a}}_{{m}_{2}}+\,\mathrm{H}.\mathrm{c}\,.\right)+h{\hat{\sigma }}_{\ell }^{x}.$$

The first term describes matter tunnelling of strength *J* between the two sites mediated by the gauge field. In the electric field basis, each tunnelling event flips the gauge field configuration ($\left\vert {-}_{\ell }\right\rangle \leftrightarrow \left\vert {+}_{\ell }\right\rangle$). The second term represents the electric field energy. It sets the cost *h* of creating or destroying an electric field line between the matter sites. Importantly, the evolution preserves the local gauge symmetry as ${\hat{H}}_{{{\mathbb{Z}}}_{2}}^{{\rm{link}}}$ commutes with the symmetry operators $[{\hat{H}}_{{{\mathbb{Z}}}_{2}}^{{\rm{link}}},{\hat{G}}_{{m}_{1}}]=[{\hat{H}}_{{{\mathbb{Z}}}_{2}}^{{\rm{link}}},{\hat{G}}_{{m}_{2}}]=0$ (Methods). For an initial state $\left\vert {\psi }_{0}\right\rangle$ that satisfies the symmetry constraint ${\hat{G}}_{{m}_{i}}\left\vert {\psi }_{0}\right\rangle ={g}_{{m}_{i}}\left\vert {\psi }_{0}\right\rangle$, the dynamics of the system remains confined to the subspace defined by the eigenvalues $({g}_{{m}_{1}},{g}_{{m}_{2}})$, where ${g}_{{m}_{i}}\in \{-1,1\}$, as ${\hat{G}}_{{m}_{i}}^{2}={\mathbb{I}}$. The values ${g}_{{m}_{i}}=-{(-1)}^{{q}_{{m}_{i}}^{{\rm{bg}}}}$ determine the background charges ${q}_{{m}_{i}}^{{\rm{bg}}}$, which are static. Electric field lines stretch between background and dynamic charges.

First, we focus on the $({g}_{{m}_{1}},{g}_{{m}_{2}})=(1,-1)$ subspace, which has a single dynamical matter excitation. For *h* = 0, the dynamics is restricted to the two-level subspace:

2 $$\left\vert \psi (t)\right\rangle =\cos (\,Jt)\left\vert {1}_{{m}_{1}},{-}_{\ell },{0}_{{m}_{2}}\right\rangle +{\mathrm{i}}\sin (\,Jt)\left\vert {0}_{{m}_{2}},{+}_{\ell },{1}_{{m}_{2}}\right\rangle ,$$

where the ket notation includes only the dynamical matter and gauge field configuration, as the background charges stay fixed. This is a manifestation of the ${{\mathbb{Z}}}_{2}$ version of Gauss’s law, as the movement of the charge is associated with the stretching (presence) or compressing (absence) of an electric field line emanating from the fixed background source ${q}_{{m}_{1}}^{{\rm{bg}}}=1$ and following the dynamical charge, which acts as a sink.

Under the full Hamiltonian in equation (1), that is, *h* ≠ 0, the gauge (${\bar{s}}_{\ell }^{x}$) and matter (${\bar{n}}_{{m}_{1}}$, ${\bar{n}}_{{m}_{2}}$) observables oscillate with the same frequency ${\Omega }_{0}=\sqrt{{J}^{2}+{h}^{2}}$, thus exhibiting correlations that confirm the intertwining of the gauge and matter fields:

3 $$\begin{array}{lll}{\overline{n}}_{{m}_{1}}(t) & \equiv & \langle {\hat{a}}_{{m}_{1}}^{\dagger }{\hat{a}}_{{m}_{1}}\rangle (t)=1-\frac{{J}^{2}}{{\Omega }_{0}^{2}}{\sin }^{2}({\Omega }_{0}t),\\ {\overline{n}}_{{m}_{2}}(t) & \equiv & \langle {\hat{a}}_{{m}_{2}}^{\dagger }{\hat{a}}_{{m}_{2}}\rangle (t)=\frac{{J}^{2}}{{\Omega }_{0}^{2}}{\sin }^{2}({\Omega }_{0}t),\\ {\overline{s}}_{\ell }^{x}(t) & \equiv & \langle {\hat{\sigma }}_{\ell }^{x}\rangle (t)=-1+\frac{2{J}^{2}}{{\Omega }_{0}^{2}}{\sin }^{2}({\Omega }_{0}t).\end{array}$$

As the electric field energy increases, stretching or compressing field lines becomes increasingly costly, which inhibits charge dynamics, the precursor to confinement in larger systems^35^.

To add another spatial dimension, we need to add more links, which in turn requires a larger qubit register. The minimal building block for 2 + 1D is realized with another qubit. Both qubits encode gauge fields and independently mediate tunnelling between two matter sites forming a loop, as shown in Fig. 1e,f. This configuration resembles a traditional Wilson loop^7^ but involves only two matter sites instead of four.

The dynamics within the ${{\mathbb{Z}}}_{2}$ loop are described by

4 $${\hat{H}}_{{{\mathbb{Z}}}_{2}}^{{\rm{loop}}}=\sum _{i=1,2}\left(\left(J{\hat{a}}_{{m}_{2}}^{\dagger }{\hat{\sigma }}_{{\ell }_{i}}^{z}{\hat{a}}_{{m}_{1}}+{\rm{H}}.{\rm{c}}.\right)+h{\hat{\sigma }}_{{\ell }_{i}}^{x}\right),$$

which retains a local symmetry generated by $${\hat{G}}_{{m}_{i}}=\,{\hat{\sigma }}_{{\ell }_{1}}^{x}{\hat{\sigma }}_{{\ell }_{2}}^{x}{\hat{P}}_{{m}_{i}}$$ for *i* ∈ {1, 2}. To ensure Gauss’s law, we could initialize the gauge fields in the electric field basis, $$\left\vert {+}_{{\ell }_{1}},{+}_{{\ell }_{1}}\right\rangle$$, $$\left\vert {-}_{{\ell }_{1}},{-}_{{\ell }_{1}}\right\rangle$$, $$\left\vert {-}_{{\ell }_{1}},{+}_{{\ell }_{1}}\right\rangle$$ or $$\left\vert {+}_{{\ell }_{1}},{-}_{{\ell }_{1}}\right\rangle$$, which are eigenstates of $${\hat{G}}_{{m}_{i}}$$. Here, unlike in the single-link case, Gauss’s law is less restrictive, which allows us to work in the magnetic field basis instead. In this basis, the gauge fields are prepared in superpositions forming maximally entangled Bell pairs, $$|{\Phi }_{{\ell }_{1}{\ell }_{2}}^{\pm }\rangle =\left(|{+}_{{\ell }_{1}},{\pm }_{{\ell }_{2}}\rangle \right.$$ $$\left.+|{-}_{{\ell }_{1}},{\mp }_{{\ell }_{2}}\rangle \right)/\sqrt{2}$$ and $$\vert {\Psi }_{{\ell }_{1}{\ell }_{2}}^{\pm }\rangle =(\left\vert {\pm }_{{\ell }_{1}},{+}_{{\ell }_{2}}\right\rangle -\left\vert {\mp }_{{\ell }_{1}},{-}_{{\ell }_{2}}\right\rangle )/\sqrt{2}$$. Note that these are the regular Bell states that we typically express in the computational basis $$|{{\Phi }}_{{\ell }_{1}{\ell }_{2}}^{\pm }\rangle =$$$$(|{\uparrow }_{{\ell }_{1}},{\uparrow }_{{\ell }_{2}}\rangle \pm |{\downarrow }_{{\ell }_{1}},{\downarrow }_{{\ell }_{2}}\rangle )/\sqrt{2}$$ and $$\vert {\Psi }_{{\ell }_{1}{\ell }_{2}}^{\pm }\rangle =(\vert {\uparrow }_{{\ell }_{1}},{\downarrow }_{{\ell }_{2}}\rangle \pm\vert {\downarrow }_{{\ell }_{1}},{\uparrow }_{{\ell }_{2}}\rangle )/\sqrt{2}$$. These states encode the presence or absence of a $${{\mathbb{Z}}}_{2}$$ magnetic flux through the surface enclosed by the two links. As the dynamical matter excitation tunnels via either link, it acquires a relative phase depending on the gauge field configuration. This results in constructive or destructive interference between the two paths, leading to the observation of the Aharonov–Bohm effect, a surface (2 + 1D) phenomena, with a phase given by

5 $${\phi }_{{\rm{AB}}}=\arccos (\langle {\hat{\sigma }}_{{\ell }_{1}}^{z}{\hat{\sigma }}_{{\ell }_{2}}^{z}\rangle ).$$

In the $\vert {\Phi }_{{\ell }_{1}{\ell }_{2}}^{\pm }\rangle$ configuration, a matter excitation acquires the same phase whether it tunnels via *ℓ*_1_ or *ℓ*_2_ (*ϕ*_AB_ = 0), indicating the absence of magnetic flux. Initializing in $\left\vert {\psi }_{0}\right\rangle =\vert {1}_{{m}_{1}},{\Phi }_{{\ell }_{1}{\ell }_{2}}^{+},{0}_{{m}_{2}}\rangle$ and setting *h* = 0, the state evolves as

6 $$\left\vert \psi (t)\right\rangle ={\cos} (2Jt)\left\vert {1}_{{m}_{1}},{\Phi }_{{\ell }_{1}{\ell }_{2}}^{+},{0}_{{m}_{2}}\right\rangle +{\mathrm{i}}{\sin} (2Jt)\left\vert {0}_{{m}_{1}},{\Phi }_{{\ell }_{1}{\ell }_{2}}^{-},{1}_{{m}_{2}}\right\rangle,$$

with tunnelling at rate 2*J*. Enforced by Gauss’s law, the ${{\mathbb{Z}}}_{2}$ charge dynamics synchronize with coherent oscillations between orthogonal Bell states, thereby producing a periodic inversion of $\langle {\hat{\sigma }}_{{\ell }_{1}}^{x}{\hat{\sigma }}_{{\ell }_{2}}^{x}\rangle$, while the ${{\mathbb{Z}}}_{2}$ magnetic flux (*ϕ*_AB_ = 0) remains zero throughout. This behaviour resembles the stretching and compression of the electric field line observed in the single-link case.

Conversely, in the $\vert {\Psi }_{{\ell }_{1}{\ell }_{2}}^{\pm }\rangle$ configuration, the matter excitation acquires phases *ϕ*_1_ or *ϕ*_2_ = *ϕ*_1_ + π when tunnelling via *ℓ*_1_ or *ℓ*_2_, resulting in *ϕ*_*AB*_ = π, and thus, magnetic flux and vison are present. This causes destructive Aharonov–Bohm interference, which inhibits tunnelling; hence, the state $\left\vert {\psi }_{0}\right\rangle =\vert {1}_{{m}_{1}},{\Psi }_{{\ell }_{1}{\ell }_{2}}^{\pm },{0}_{{m}_{2}}\rangle$ remains frozen for all time.

## Initializing and measuring the single-link hybrid system

In the experiment, the matter sites were encoded in harmonic oscillators formed by normal modes of vibration of a one- or two-ion crystal. We prepared these oscillators with *k* quanta $\left\vert {k}_{{m}_{i}}\right\rangle$. Moreover, each gauge field was represented by a qubit encoded in the internal level structure of each ion in the crystal. The qubit states were given by two electronic levels, $\left\vert {\downarrow }_{\ell }\right\rangle$ and $\left\vert {\uparrow }_{\ell }\right\rangle$. Experimental details of our trapped-ion system are provided in Methods.

We first focused on realizing the dynamics of a single link using a single ion, as shown in Fig. 1c,d, where the LGT degrees of freedom are encoded in its qubit *ℓ* and two motional modes, *m*_1_ and *m*_2_. The quantum processor operated in a hybrid mode: digital for state preparation, error mitigation and read-out and analogue during gauge-invariant tunnelling. By applying the tunnelling interaction for a variable duration, we could simulate the time evolution of any initialized state.

To synthesize the first term in equation (1), the matter tunnelling mediated by the gauge field, we employed the scheme proposed in ref. ^42^, which has previously been used to generate single-mode generalized squeezing^43,44^. This scheme combines two qubit-state-dependent forces, each addressing a different oscillator: one detuned by *Δ* from oscillator *m*_1_, the other by *Δ* from oscillator *m*_2_. As the forces are conditioned on ${\hat{\sigma }}_{\ell }^{x}$ and ${\hat{\sigma }}_{\ell }^{y}$, their non-commutativity gives rise to an effective resonant tunnelling interaction conditioned on ${\hat{\sigma }}_{\ell }^{z}\propto [{\hat{\sigma }}_{\ell }^{x},{\hat{\sigma }}_{\ell }^{y}]$. The ability to engineer qubit-state-dependent forces with arbitrary conditioning enabled complete control over the state-dependence of the tunnelling term in equation (1). Furthermore, this scheme relies on smoothly switching the interaction on and off over a duration *t*_R_, thus setting a lower bound on the pulse width: *t* ≥ *t*_R_. A detailed explanation of this scheme is provided in Methods. Alternative methods to create state-conditioned tunnelling exist, for example, refs. ^45,46^; however the resulting Hamiltonian may not be gauge-invariant.

To initialize the hybrid system in $\left\vert {\psi }_{0}\right\rangle =\left\vert {1}_{{m}_{1}},{-}_{\ell },{0}_{{m}_{2}}\right\rangle$, we first cooled the vibrational modes close to their ground state ($\bar{n}\approx 0$) via Doppler and resolved-sideband cooling. The internal state of the ion was then optically pumped to $\left\vert {\downarrow }_{\ell }\right\rangle$. A resonant anti-Jaynes–Cumming interaction (blue sideband pulse) on *m*_1_ prepared $\left\vert {1}_{{m}_{1}},{\uparrow }_{\ell },{0}_{{m}_{2}}\right\rangle$, which was followed by a π/2 qubit rotation to produce $\left\vert {1}_{{m}_{1}},{-}_{\ell },{0}_{{m}_{2}}\right\rangle$ (Methods).

The read-out sequence for the hybrid state depends on the observable. We measured the gauge field state (${\bar{s}}_{\ell }^{x}$) by applying a π/2 qubit rotation followed by fluorescence detection. To efficiently read out matter site expectation values (${\bar{n}}_{{m}_{1}}$, ${\bar{n}}_{{m}_{2}}$), we restricted the tomography to the subspace with at most one excitation per site. We performed fluorescence detection and post-selected on the dark state, thus tracing out the qubit–oscillator entanglement. We then read out the matter state via the qubit by applying a blue sideband pulse on the respective oscillator and performed a final fluorescence detection (Methods or ref. ^45^).

## ${\pmb{\mathbb{Z}}}_{\bf{2}}$ link tunnelling

**Fig. 2:**
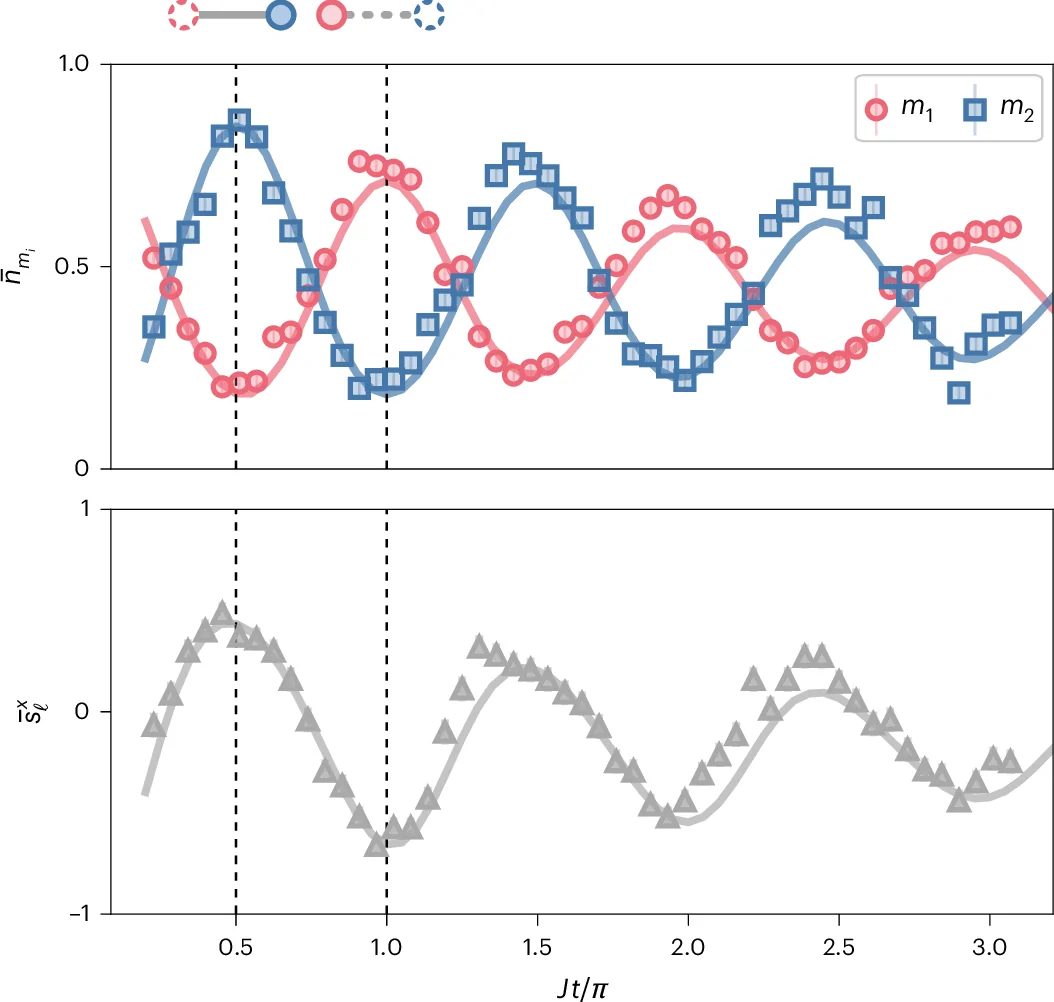
Matter excitation dynamically tunnelling between matter sites *m*_1_ and *m*_2_ as a function of the evolution time of the Hamiltonian in equation (1) with *h* = 0. We prepared the initial state $\left\vert {1}_{{m}_{1}},{-}_{\ell },{0}_{{m}_{2}}\right\rangle$ and measured the observables corresponding to the gauge field ${\bar{s}}_{\ell }^{x}$ and matter occupation ${\bar{n}}_{{m}_{1}}$ and ${\bar{n}}_{{m}_{2}}$. When the matter tunnelled between the sites, the gauge field inverted. The insets above the dotted lines illustrate the system configuration at different time points, corresponding to the states $\left\vert {0}_{{m}_{1}},{+}_{\ell },{1}_{{m}_{1}}\right\rangle$ and $\left\vert {1}_{{m}_{1}},{-}_{\ell },{0}_{{m}_{1}}\right\rangle$. Solid lines show simulations with independently estimated parameters, except for the tunnelling rate *J*, which was adjusted by 12% to match the experimental timescale. This correction reflects a discrepancy between the expected drive strength and the measured dynamics and is left for future investigation. We extracted a tunnelling rate *J*/π = 1.47 kHz. Owing to the required pulse ramping, the minimum possible interaction duration (full-width at half-maximum) is given by 2*t*_R_*J*/π = 0.24 instead of 0. We show the mean of the quantum projection measurements. Error bars (68% confidence intervals) are omitted when smaller than the markers. Gauge field measurements used 300 shots per point. Post-selection during the conditional read-out reduced the effective sample size for matter field measurements to ≥298 shots, depending on the data point. Source data

We first consider the case of vanishing electric field energy, that is, *h* = 0 in equation (1). After initializing the system in the state $\left\vert {\psi }_{0}\right\rangle =\left\vert {1}_{{m}_{1}},{-}_{\ell },{0}_{{m}_{2}}\right\rangle$, we evolved it under gauge-invariant tunnelling alone and performed the read-out. Figure 2 shows the experimentally measured expectation values of the gauge-invariant observables ${\bar{n}}_{{m}_{1}}(t)$, ${\bar{n}}_{{m}_{2}}(t)$ and ${\bar{s}}_{\ell }^{x}(t)$ alongside values from numerical simulations. The data demonstrate coherent charge tunnelling synchronized with the stretching and compressing of the electric field lines. The agreement with numerical predictions confirms our understanding of how imperfections in the quantum processor affect the dynamics. The correlation between the three experimentally measured observables and the agreement between experimental and numerical simulation data are quantified in Methods for all data presented in this and subsequent figures. Unlike equation (3), these predictions incorporate an initial thermal occupation ${\bar{n}}_{{m}_{1}}(0)={\bar{n}}_{{m}_{2}}(0)=0.1$ due to imperfect ground-state cooling as well as motional heating rates ${\dot{n}}_{{m}_{1}}$ = 300 quanta per second and ${\dot{\bar{n}}}_{{m}_{2}}$ = 5 quanta per second. These independently calibrated decoherence sources explain the reduced and slowly decaying contrast compared with the idealized case, which we show in Methods. Although these errors weakly break gauge symmetry, we still observed correlated dynamics for the matter and gauge fields.

Other sources of error include uncompensated a.c. Stark shifts and magnetic field fluctuations, which introduce a dephasing term proportional to ${\hat{\sigma }}_{\ell }^{z}$ that could also break gauge invariance. To mitigate these effects, we split the tunnelling dynamics into two parts, interleaved with a qubit-echo refocusing sequence^47^.

## ${\pmb{\mathbb{Z}}}_{\bf{2}}$ link tunnelling and electric field energy

**Fig. 3:**
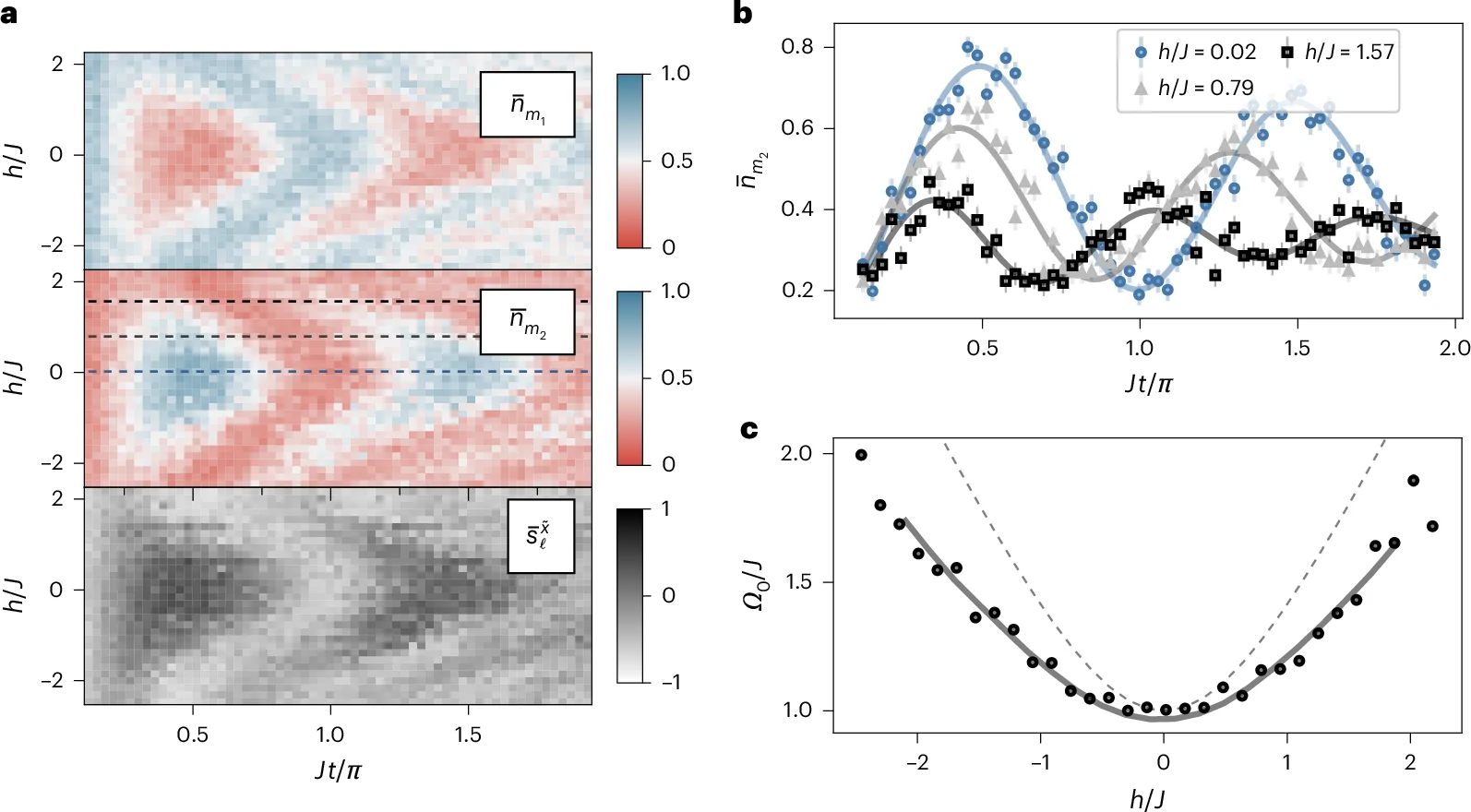
Real-time dynamics of the observables ${\bar{n}}_{{m}_{1}}(t)$, ${\bar{n}}_{{m}_{2}}(t)$ and ${\bar{s}}_{\ell }^{\tilde{x}}(t)$ evolving under the full ${{\mathbb{Z}}}_{2}^{{\rm{link}}}$ Hamiltonian given by equation (1) but in the rotated basis. **a**, In the two-dimensional scans, we found clear correlations between matter (${\bar{n}}_{{m}_{1}}$, ${\bar{n}}_{{m}_{2}}$) and gauge field observable ${\bar{s}}_{\ell }^{\tilde{x}}\equiv {\bar{s}}_{\ell }^{z}$, where $\tilde{x}$ takes the role of the *x* observable in the transformed basis. **b**, Three slices of ${\bar{n}}_{{m}_{2}}$ at electric field settings *h*/*J* = {0.02, 0.79, 1.57}, as indicated by the dashed lines in **a**. We show the mean of the quantum projection measurements. Error bars (68% confidence intervals) are omitted when smaller than the markers. Gauge field measurements used 300 shots per point. Post-selection during the conditional read-out reduced the effective sample size for matter field measurements to ≥108 shots, depending on the data point. We fitted a decaying sinusoid to each slice and obtained the oscillation frequency *Ω*_0_/*J* versus field energy *h*/*J*. Errors were inferred from the estimated covariance matrix of the fit. **c**, Extracted oscillation frequency *Ω*_0_/*J* versus the field energy *h*/*J*. Errors were inferred from the estimated covariance matrix of the fit. We measured a tunnelling rate *J*/π = *Ω*_0_(0)/π = 1.510(6) kHz. The experimental data agree with the numerical simulation made using independent parameter estimates (solid line). Within the ${{\mathbb{Z}}}_{2}$ LGT framework, where *Ω*_0_ is expected to follow the analytic prediction ${\Omega }_{0}=J\sqrt{1+{(h/J)}^{2}}$, the observed corrections can be interpreted as a reduction of the electric field, *h*_eff_ < *h*, in the realized quantum simulation. Source data

Next, we simulated the dynamics in equation (1) with electric field energy ∣*h*∣ > 0. To minimize the technical overhead, we implemented the tunnelling conditioned on ${\hat{\sigma }}_{\ell }^{x}$, which allowed us to add the electric field term by introducing a qubit frequency offset $h{\hat{\sigma }}_{\ell }^{z}$. This yielded the Hamiltonian in equation (1) but in a rotated basis, where ${\hat{\sigma }}_{\ell }^{\tilde{z}}={\hat{\sigma }}_{\ell }^{x}\,\mathrm{and}\,{\hat{\sigma }}_{\ell }^{\tilde{x}}={\hat{\sigma }}_{\ell }^{z}$ (Methods). We investigated the dynamics of the matter excitation and the correlated gauge field while keeping *J* constant and varying *h*, as shown in Fig. 3a.

We extracted *Ω*_0_(*h*) by fitting a decaying sinusoid to the time evolution of ${\bar{n}}_{{m}_{2}}(t)$, as shown in Fig. 3b,c. Consistent with the prediction of equation (3), we observed that increasing ∣*h*∣ enhanced the tunnelling rate *Ω*_0_ while suppressing the oscillation amplitude. The extracted values of *Ω*_0_ agree with numerical simulations but deviate from the analytic theory, indicating that the effective electric field term is smaller than expected.

The observed dynamics indicates that there was a gradual transition from a tunnelling-dominated regime to one where the gauge field becomes effectively pinned, inhibiting matter tunnelling, as ∣*h*/*J*∣ increases. This is a direct manifestation of the intertwining of charge and gauge field properties: stretching or compressing the electric field line becomes increasingly costly, and hence, charge tunnelling is suppressed. In larger lattices, ∣*h*∣ > 0 would lead to a linearly rising potential between a pair of charges, resulting in a Wannier–Stark confinement^35^ analogous to the quark–antiquark confinement in mesons^35^.

## Aharonov–Bohm effect on a ${\pmb{\mathbb{Z}}}_{\bf{2}}$ loop

We introduced a second spatial dimension to our system to observe real-time dynamics in the ${{\mathbb{Z}}}_{2}$ loop shown in Fig. 1e,f and described by the gauge-invariant Hamiltonian in equation (4). This was achieved by adding a second ion to the crystal, which introduced another gauge field that independently mediated tunnelling between the two matter sites. The matter sites (*m*_1_, *m*_2_) correspond to two of the normal motional modes of the two-ion crystal. To simulate the effect of the magnetic flux piercing the loop, we initialized the gauge fields (*l*_1_, *l*_2_) in the magnetic field basis. Using the digital control capabilities of the trapped-ion hybrid device, we prepared two initial entangled gauge field configurations, $\left\vert {\psi }_{0}\right\rangle \in \{\vert {1}_{{m}_{1}},{\Phi }_{{\ell }_{1}{\ell }_{2}}^{+},{0}_{{m}_{2}}\rangle ,\vert {1}_{{m}_{1}},{\Psi }_{{\ell }_{1}{\ell }_{2}}^{+},{0}_{{m}_{2}}\rangle\}$, where ${\Phi }_{{\ell }_{1}{\ell }_{2}}^{+}$ and ${\Psi }_{{\ell }_{1}{\ell }_{2}}^{+}$ are Bell states generated through a sequence of single- and two-qubit entangling gates (Methods). These gauge field states correspond to effective magnetic fluxes of 0 and π through the loop, respectively.

We then applied the analogue tunnelling interaction to observe the real-time evolution of the ${{\mathbb{Z}}}_{2}$ loop under the gauge-invariant Hamiltonian in equation (4) with *h* = 0. Following this, we measured the system observables: the matter site occupations ${\bar{n}}_{{m}_{1}}(t)$ and ${\bar{n}}_{{m}_{2}}(t)$ and the gauge field correlator ${\bar{s}}_{{\ell }_{1}{\ell }_{2}}^{xx}(t)=\langle {\hat{\sigma }}_{{\ell }_{1}}^{x}{\hat{\sigma }}_{{\ell }_{2}}^{x}\rangle$, using the same methods described for the ${{\mathbb{Z}}}_{2}$ link simulation.

**Fig. 4:**
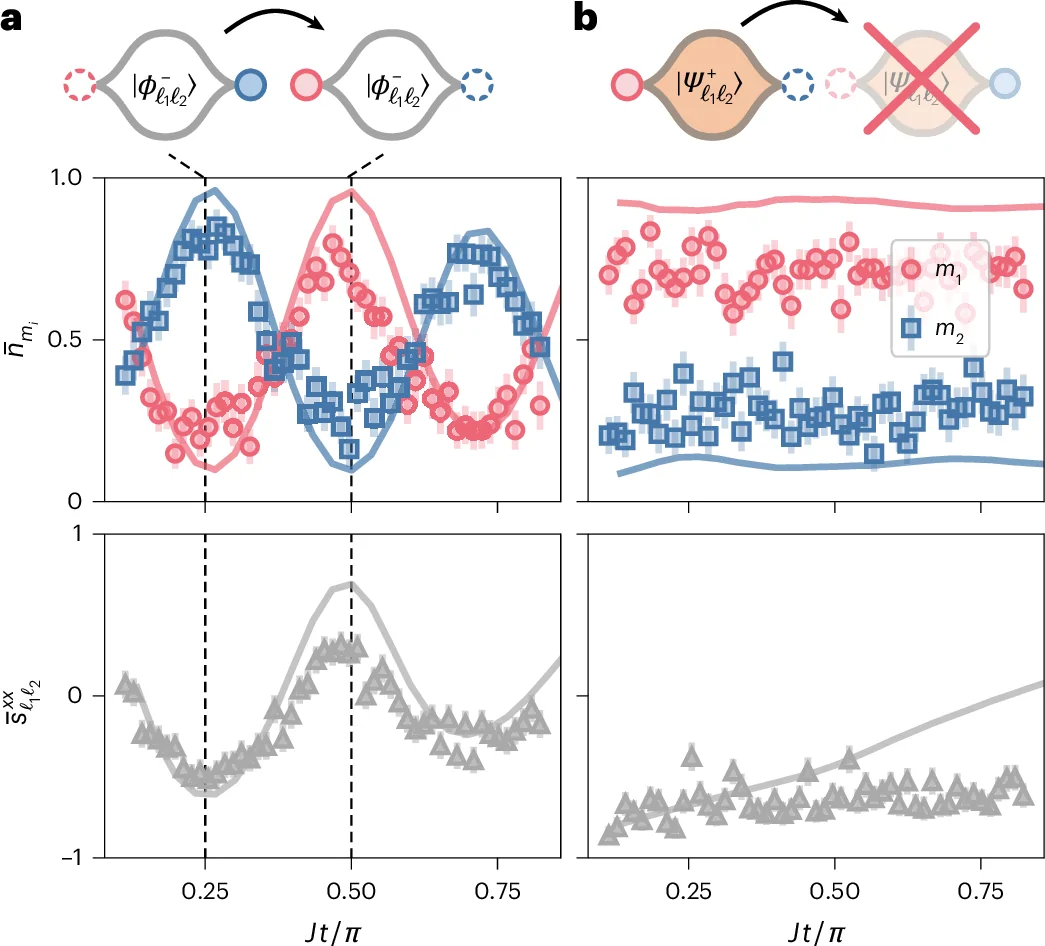
Tunnelling of matter particles under Aharonov–Bohm interference in the ${{\mathbb{Z}}}_{2}$ loop (equation (4)). **a**,**b**, By preparing initial states with entangled gauge fields, we emulated a magnetic flux and encoded an Aharonov–Bohm phase *ϕ*_AB_ via the dynamical degrees of freedom of the gauge field. We tracked the expectation values ${\bar{n}}_{{m}_{1}}$, ${\bar{n}}_{{m}_{2}}$ and ${\bar{s}}_{{\ell }_{1}{\ell }_{2}}^{xx}$. **a**, For the initial state $\vert {1}_{{m}_{1}},{\Phi }_{{\ell }_{1}{\ell }_{2}}^{+},{0}_{{m}_{2}}\rangle$, corresponding to zero magnetic flux (*ϕ*_AB_ = 0), we observed coherent matter tunnelling correlated with ${\bar{s}}_{{\ell }_{1}{\ell }_{2}}^{xx}$. The insets above the dashed lines show the intermediate states $\vert {0}_{{m}_{1}},{\Phi }_{{\ell }_{1}{\ell }_{2}}^{-},{1}_{{m}_{2}}\rangle$ and $\vert {1}_{{m}_{1}},{\Phi }_{{\ell }_{1}{\ell }_{2}}^{+},{0}_{{m}_{2}}\rangle$. Supplementary data in ‘Characterizing the flux and parity oscillations of Bell states’ confirm oscillations between these configurations (Methods). We extracted a tunnelling rate *J*/π = 0.35 kHz. **b**, For $\vert {1}_{{m}_{1}},{\Psi }_{{\ell }_{1}{\ell }_{2}}^{+},{0}_{{m}_{2}}\rangle$, shown in the inset, a π flux (*ϕ*_AB_ = π, orange shading) was introduced, leading to destructive Aharonov–Bohm interference such that tunnelling was inhibited. All observables remained constant. Solid lines are numerical simulations using independently estimated parameters including oscillator heating and qubit decoherence, except for the tunnelling rate *J*, which was floated to match the experimental timescale. We show the mean of the quantum projection measurements. Error bars (68% confidence intervals) are omitted when smaller than the markers. Post-selection during the conditional read-out reduced the effective sample size to ≥203 shots per point for gauge field measurements and to ≥64 shots for matter field measurements, depending on the data point. Source data

These observables, shown in Fig. 4, track the correlated dynamics between matter excitations and the entangled gauge fields. Additionally, we verify in Methods that ${\bar{s}}_{{\ell }_{1}{\ell }_{2}}^{zz}(t)=\langle {\hat{\sigma }}_{{\ell }_{1}}^{z}{\hat{\sigma }}_{{\ell }_{2}}^{z}\rangle$ remained constant, confirming that the flux and corresponding Aharonov–Bohm phase ${\phi }_{{\rm{AB}}}=\arccos (\langle {\hat{\sigma }}_{{\ell }_{1}}^{z}{\hat{\sigma }}_{{\ell }_{2}}^{z}\rangle)$ were conserved throughout the evolution, aside from a gradual amplitude decay caused by decoherence.

When the gauge fields were initialized in the Bell state $\vert {\Phi }_{{\ell }_{1}{\ell }_{2}}^{+}\rangle$, corresponding to zero magnetic flux (*ϕ*_AB_ = 0), no vison was present. In this scenario (Fig. 4a), the matter excitation tunnelled freely, with dynamics intertwined with the gauge field, and oscillated between $\vert {1}_{{m}_{1}},{\Phi }_{{\ell }_{1}{\ell }_{2}}^{+},{0}_{{m}_{2}}\rangle \leftrightarrow\vert {0}_{{m}_{1}},{\Phi }_{{\ell }_{1}{\ell }_{2}}^{-},{1}_{{m}_{2}}\rangle$, as described by equation (6). By contrast, the Bell state $\vert {\Psi }_{{\ell }_{1}{\ell }_{2}}^{+}\rangle$ corresponded to a flux *ϕ*_AB_ = π, which indicates the presence of a vison. This flux induced destructive interference that suppressed tunnelling (Fig. 4b), thereby effectively freezing the system in the initial configuration.

## More results and future work

Our experimental findings demonstrate the role of gauge field entanglement in Aharonov–Bohm interference and the weaving of flux and charge in a LGT quantum simulator. The advances presented here highlight the potential of the hybrid qubit–oscillator encoding for simulating LGTs in synthetic dimensions. Building on this, we outline three directions for future exploration. First, introducing an electric field energy (∣*h*∣ > 0) to the loop would lead to a three-way competition between the Aharonov–Bohm effect, tunnelling and the field energy itself. This interplay would offer a tunable setting for exploring interference-driven localization and its breakdown in a gauge-invariant context. We provide numerical simulations of the ideal dynamics under this extra interaction (Methods).

Second, the bosonic nature of the matter sites, encoded in the harmonic oscillators, enables richer matter dynamics that are not constrained by the Pauli exclusion principle. Beyond the single-phonon matter excitations discussed so far, this approach would also allow the exploration of superpositions of Fock states with well-defined ${{\mathbb{Z}}}_{2}$ charges. In particular, we focused on squeezed matter states, which we investigated both experimentally and numerically, as detailed in ‘Further investigations and future work’ in Methods. This direction is promising, as the Hilbert space dimension grows with the squeezing magnitude, potentially enabling classically intractable real-time dynamics without requiring large lattice sizes. Additionally, it allows for characterizations using phase-space methods, including direct measurement of the characteristic function^43,48,49^.

**Fig. 5:**
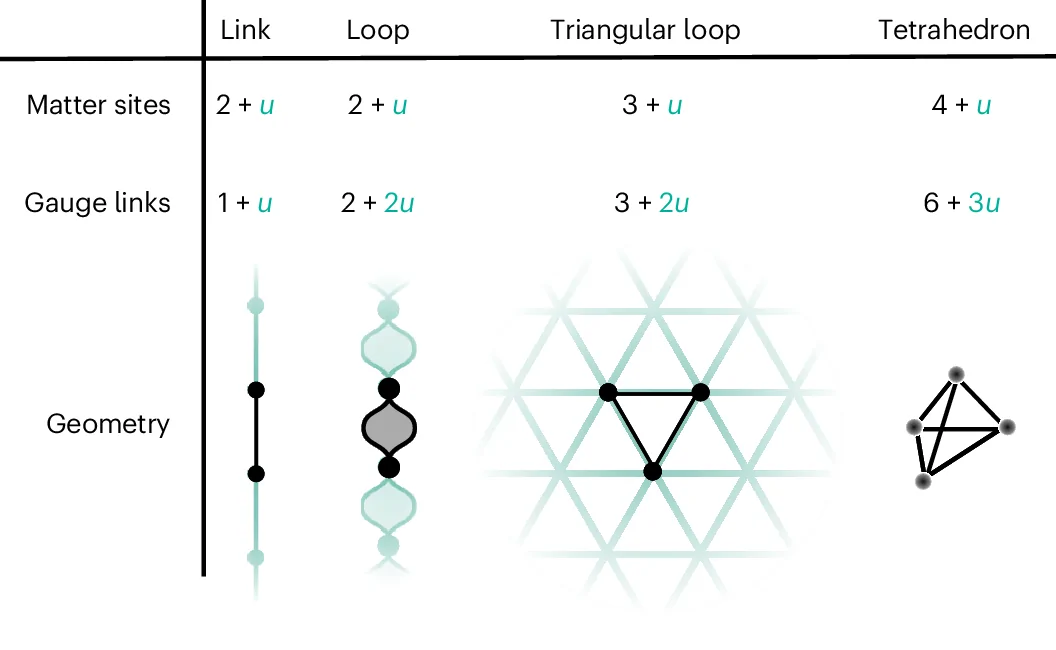
Resource requirements for elementary building blocks. The link and loop blocks, which have been realized in this work, as well as the triangular loop and tetrahedron blocks, which require only a modest increase in register size, are shown in black. The lattices formed by concatenating these blocks are illustrated in green. The resource scaling of a lattice formed by *u* + 1 blocks is also indicated.

Finally, synthetic dimensions enable the exploration of LGTs in complex geometries and provide a path to larger systems. The essential ingredients for this architecture—parametric mode coupling, Bell-state flux encoding and preparation of non-trivial oscillator states—are all demonstrated in the present work. Together with single-ion addressing, which is a standard capability in trapped-ion platforms, these ingredients provide a straightforward scaling strategy based on selectively resolving qubit–oscillator subsystems in a long ion chain, thereby effectively mapping a one-dimensional ion chain onto new synthetic dimensions and geometries. By engineering tunnelling between selected motional modes (matter sites) and selected ions (links), one can then realize chains of connected loops^50^, triangular loops^51^ and tetrahedral lattices, the simplest three-dimensional 3 + 1D LGT, all with modest experimental resources (Fig. 5). Although ultimately limited by mode crowding, this approach would allow access to moderately large systems. As a further extension, refs. ^35,50^ propose synthetic dimensional reduction via dimerized vibrational modes with locally engineered trap frequencies, thereby grouping modes into independent matter sites. Each site would then be coupled to a pair of gauge qubits via state-dependent parametric tunnelling to ensure local gauge invariance.

In conclusion, we have experimentally demonstrated a ${{\mathbb{Z}}}_{2}$ LGT on a single link, thereby encoding dynamical matter and gauge fields on an equal footing within a hybrid qubit–oscillator trapped-ion system. We employed digital techniques to prepare initial states and read out observables, and an analogue evolution simulated the real-time dynamics. Using synthetic dimensions, we extended this to an elementary 2 + 1D geometry, formed by two qubits and two harmonic oscillators on a two-ion crystal, thus achieving a quantum simulation of the real-time dynamics of a ${{\mathbb{Z}}}_{2}$ loop. By preparing entangled gauge field states corresponding to effective magnetic fluxes of 0 and π, we observed the Aharonov–Bohm effect. Matter tunnelling was inhibited for *ϕ*_AB_ = π, thus confirming the presence of a vison, whereas for *ϕ*_AB_ = 0, tunnelling remained uninhibited.

We have outlined several directions for extending this work: probing the competition between magnetic flux and electric field energy, exploring bosonic matter dynamics and engineering more complex lattice geometries, which would be enabled by the digital–analogue control and qubit–oscillator architecture of the hybrid processor. More broadly, these capabilities may provide a rich foundation for further theoretical and experimental progress, thereby paving the way for the exploration of new states of matter and phase transitions in larger Hilbert spaces with flexible connectivity that is beyond the reach of classical methods.

## Methods

### Encoding the LGT in a hybrid qubit–oscillator system

As proposed in ref. ^35^ and discussed in the main text, we encoded the gauge fields and matter sites in distinct components of a hybrid qubit–oscillator system realized with trapped ions. The trapped-ion system used in this work is described in detail in refs. ^52–54^. The gauge field is represented by a qubit formed from two internal electronic states of a single ^88^Sr^+^ ion:

7 $$\left\vert {\downarrow }_{\ell }\right\rangle \equiv \left\vert 5{S}_{1/2},{m}_{j}=-1/2\right\rangle ,\quad \left\vert {\uparrow }_{\ell }\right\rangle \equiv \left\vert 4{D}_{5/2},{m}_{j}=-3/2\right\rangle .$$

The matter sites are encoded in the normal modes of the ion crystal. To represent a link, we use a single ion but require only two of its three available motional modes: (1) the axial mode with *f*_ax-ip_ = 1.2 MHz, heating rate ${\dot{\bar{n}}}_{\mathrm{ax-ip}}$ = 300 quanta per second and coherence time *t*_c,ax-ip_ = 1.7 ms; (2) the lower radial mode with *f*_lr-ip_ = 1.5 MHz, heating rate ${\dot{\bar{n}}}_{\mathrm{lr-ip}}$ = 5 quanta per second and coherence time *t*_c,lr-ip_ = 2.3 ms and (3) the upper radial mode with *f*_ur-ip_ = 1.6 MHz, heating rate ${\dot{\bar{n}}}_{\mathrm{ur-ip}}$ = 17 quanta per second and coherence time *t*_c,ur-ip_ = 3.3 ms. The specific modes used vary across the experiments presented in this article and are listed in Supplementary Table I. For the loop configurations, we trapped a two-ion crystal, which introduced another qubit to encode the second gauge field. The collective motion of the two ions hybridized to form six normal modes, from which we selected two of the in-phase (ip) modes to represent the matter sites.

We coherently manipulated the qubit–oscillator system using a 674-nm laser with a waist size of 20 μm at the ion. This laser drove both the qubit transition and the qubit–oscillator interactions.

### Microscopic interaction creating the gauge–matter tunnelling

#### Link

In this section, for convenience, we describe the microscopic interaction in terms of qubits (used to encode the gauge fields) and motional harmonic oscillators (used to encode the matter sites). To synthesize a tunnelling interaction conditioned on the qubit (gauge field) state (equation (1)), we followed the theoretical proposal presented in ref. ^42^, which realizes a universal set of qubit-conditioned continuous-variable quantum computing interactions. We have previously used this technique to demonstrate qubit-conditioned generalized single-mode squeezing^43,44^. We extended the same approach to engineer a qubit-conditioned oscillator–oscillator interaction, thereby enabling tunnelling conditioned on the qubit state.

We created two-qubit-state-dependent forces (SDFs) via the Mølmer–Sørensen scheme using two bichromatic fields. Each SDF was detuned by *Δ* from one of the motional modes and has an SDF drive strength of *Ω*_*i*_. The Mølmer–Sørensen scheme allowed us to control the qubit-conditioning (${\hat{\sigma }}_{\ell }^{i}$) to *i* ∈ {*x*, *y*, *z*} (refs. ^55,56^), thereby enabling us to set the SDFs such that they had non-commuting qubit operators. The resulting microscopic Hamiltonian, expressed in the interaction picture and after applying the rotating-wave approximation, is given by

8 $$\begin{array}{lll}{\hat{H}}_{\mathrm{micro}} & = & \frac{\hslash {\varOmega }_{1}}{2}({\hat{a}}_{{m}_{1}}{{\rm{e}}}^{-{\rm{i}}\varDelta t}+{\hat{a}}_{{m}_{1}}^{\dagger }{{\rm{e}}}^{{\rm{i}}\varDelta t}){\hat{\sigma }}_{\ell }^{i}{\mathbb{I}}_{{m}_{2}}\\ & & +\frac{\hslash {\varOmega }_{2}}{2}{\mathbb{I}}_{{m}_{1}}{\hat{\sigma }}_{\ell }^{\,j}({\hat{a}}_{{m}_{2}}{{\rm{e}}}^{-{\rm{i}}\varDelta t}+{\hat{a}}_{{m}_{2}}^{\dagger }{{\rm{e}}}^{{\rm{i}}\varDelta t}),\end{array}$$

where *i*, *j* ∈ {*x*, *y*, *z*}.

The effective Hamiltonian governing the evolution of the system is given by the leading-order term in the Magnus expansion:

9 $$\begin{array}{lll}{\hat{H}}_{{{{{\mathbb{Z}}}}}_{2}}^{\mathrm{link}} & = & \frac{\hslash {\varOmega }_{\mathrm{eff}}}{2}\overbrace{[{\hat{\sigma }}_{\ell }^{i},{\hat{\sigma }}_{\ell }^{\,j}]}^{2{\varepsilon }_{ijk}{\hat{\sigma }}_{\ell }^{k}}({\hat{a}}_{{m}_{1}}{\hat{a}}_{{m}_{2}}^{\dagger }+{\hat{a}}_{{m}_{1}}^{\dagger }{\hat{a}}_{{m}_{2}}),\\ {\varOmega }_{\mathrm{eff}} & = & \frac{{\varOmega }_{1}{\varOmega }_{2}}{2\varDelta },\end{array}$$

where *k* ∈ {*x*, *y*, *z*}. Besides this leading term, several other effects need to be considered. First, each SDF gives rise to a geometric phase and circular excursions in phase space. By choosing a sufficiently long ramp duration, *t*_ramp_ ≫ 2π/*Δ*, the phase-space excursions can be suppressed and made negligible. Second, the geometric phase for a single qubit is global and, thus, physically irrelevant. However, as we discuss in ‘Loop: generalizing the SDF method to two ions’, this is no longer true for the loop case, which is encoded using two qubits.

Setting *ℏ* = 1, *i* = *x* and *j* = *y* results in *k* = *z*, and associating *Ω*_eff_ = *J*, we obtained the first term in equation (1). This setting was used to simulate the results presented in Figs. 2 and 4. By switching the indices *i* = *y* and *j* = *x*, we conjugated the sign of the spin operator $-{\hat{\sigma }}_{\ell }^{z}$. This property was used to embed the interaction in a spin-echo sequence to refocus the noise without cancelling the tunnelling interaction.

To conduct the experiment illustrated in Fig. 3, we set *i* = *z* and *j* = *y*, which resulted in tunnelling conditioned on *k* = *x*. We then introduced the electric field energy by detuning all applied optical fields relative to the qubit by *h*, giving rise to

10 $${H}_{{{\mathbb{Z}}}_{2}}^{{\rm{link}}^{\prime} }=\left(J{\hat{a}}_{{m}_{1}}^{\dagger }{\hat{\sigma }}_{\ell }^{x}{\hat{a}}_{{m}_{2}}+{\rm{H}}.{\rm{c}}.\right)+h{\hat{\sigma }}_{\ell }^{z},$$

which corresponds to the full Hamiltonian in equation (1) in the Hadamard basis ${\hat{\sigma }}_{\ell }^{\tilde{z}}={\hat{\sigma }}_{\ell }^{x}$ and ${\hat{\sigma }}_{\ell }^{\tilde{x}}={\hat{\sigma }}_{\ell }^{z}$. The symmetry operators in this basis read ${\hat{\tilde{G}}}_{{m}_{i}}={\hat{\sigma }}_{\ell }^{\tilde{x}}{\hat{P}}_{{m}_{i}}={\hat{\sigma }}_{\ell }^{z}{\hat{P}}_{{m}_{i}}$.

#### Loop: generalizing the SDF method to two ions

Adding a second ion to the crystal introduced a challenge: both qubits coupled simultaneously to the driving field (${\hat{\sigma }}_{\ell }^{k}\to {\hat{S}}_{{\ell }_{1}{\ell }_{2}}^{k}={\hat{\sigma }}_{{\ell }_{1}}^{k}\otimes {{\mathbb{I}}}_{2}+{{\mathbb{I}}}_{2}\otimes {\hat{\sigma }}_{{\ell }_{2}}^{k}$). When we applied our non-linear oscillator interactions to two ions, SDFs detuned by *Δ* from their respective motional modes generated a geometric phase ${\varphi }_{\mathrm{geom}}\propto {({\varOmega }_{i })}^{2}/\varDelta$. For a single ion, this phase was global and could be neglected. However, with two ions, it led to unwanted qubit–qubit entanglement. To cancel this effect, we split the interaction pulse into two segments and reversed the detuning in the second pulse, *Δ* → −*Δ*. This reversal changed the direction of the phase-space trajectory, thereby cancelling the accumulated geometric phase. Simultaneously, we adjusted the interaction phase to ensure that the tunnelling contributions added constructively. To suppress any residual asymmetry caused by the detuning reversal, we embedded this two-pulse sequence within each arm of a qubit-echo Ramsey sequence, such that each arm consisted of two pulses: one with detuning *Δ* and one with −*Δ*.

### State preparation and measuring the observables

#### Link

**Extended Data Fig. 1:**
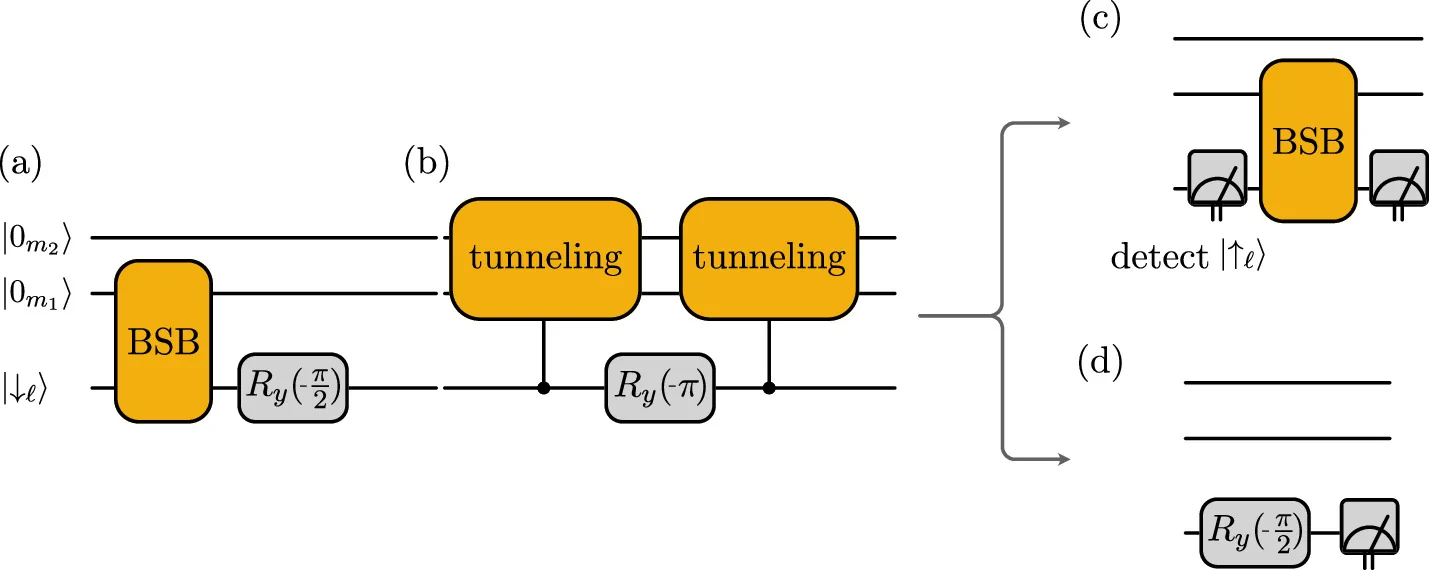
Circuit diagram for ${{\mathbb{Z}}}_{2}$ link. (**a**) Preparing the initial state. A blue-sideband (BSB) *π* pulse prepares a single excitation in the mode it is applied to. After the following *π*/2-pulse on the qubit we prepare the state $\left\vert {1}_{{m}_{1}},{-}_{\ell },{0}_{{m}_{2}}\right\rangle$. (**b**) The ${{\mathbb{Z}}}_{2}$ interaction (conditioned on ${\hat{\sigma }}_{\ell }^{z}$ represented by a dot) is applied in two blocks split by a *π*-pulse. This additional *π*-pulse protects us against external noise $\propto {\hat{\sigma }}_{\ell }^{z}$ (spin-echo sequence). We adjust the phase of the second ${{\mathbb{Z}}}_{2}$ interaction such that the conditioning is $\propto -{\hat{\sigma }}_{\ell }^{z}$ such that the echo does not affect the main interaction. Finally, we apply the readout sequence. Depending on whether we read out motion or spin we apply sequence (**c**) or (**d**). (**c**) Readout sequence for the motion ${\bar{n}}_{{m}_{1}}$ and ${\bar{n}}_{{m}_{2}}$, respectively. We first perform a mid-circuit detection on the spin. This yields $\left\vert {\uparrow }_{\ell }\right\rangle$ in 50% of the cases which we post-select. We can then apply a final ${{\rm{B}}SB}_{{m}_{i}}$ *π*-pulse to probe for an excitation in this mode. (**d**) Readout sequence for the spin ${\bar{s}}_{\ell }^{x}=\langle {\hat{\sigma }}_{\ell }^{x}\rangle$. A single *π*/2 rotation is followed by the projective measurement.

All motional modes used for the encoding were prepared in thermal states with mean occupation $\bar{n}\approx 0.1$, which is close to but not exactly in the ground state, using Doppler and sideband cooling. To keep the notation simple, we represent the states as pure states, that is, $\left\vert {0}_{{m}_{1}},{\downarrow }_{\ell },{0}_{{m}_{2}}\right\rangle$, while fully incorporating the effects of finite temperature in the numerical simulations. A quantum circuit diagram of the whole experiment is depicted in Extended Data Fig. 1. We applied a blue sideband π pulse to add a single phonon to one of the matter sites, thereby preparing the state $\left\vert {1}_{{m}_{1}},{\uparrow }_{\ell },{0}_{{m}_{2}}\right\rangle$. Then, we prepared the gauge field by applying a carrier π/2-pulse to reach the desired initial state $\left\vert {1}_{{m}_{1}},{-}_{\ell },{0}_{{m}_{1}}\right\rangle$. For the read-out, the sequence depends on the to-be-measured observable. For the gauge field observable ${\bar{s}}_{\ell }^{x}$, we applied another π/2 pulse and measured the spin in the computational basis. For the matter sites, we first performed a midcircuit measurement of the spin, which collapsed with equal probability to $\left\vert {\uparrow }_{\ell }\right\rangle$ and $\left\vert {\downarrow }_{\ell }\right\rangle$ and disentangled the spin from the oscillator. The qubit measurement was realized by observing the presence ($\left\vert {\downarrow }_{\ell }\right\rangle$) or absence ($\left\vert {\uparrow }_{\ell }\right\rangle$) of scattered light. Only for the absence of scattered light $\left\vert {\uparrow }_{\ell }\right\rangle$ was this process non-destructive to the motional state of the ion, which encoded the matter sites. Hence, we selected only the $\left\vert {\uparrow }_{\ell }\right\rangle$ outcomes. We then applied another blue sideband resonant with mode 1 (mode 2) to measure ${\bar{n}}_{{m}_{1}}$ (${\bar{n}}_{{m}_{2}}$). This read-out process assumed that the population never leaks to Fock states $\left\vert n\ge 2\right\rangle$. This is not necessarily a good assumption as the axial motional mode has a large heating rate with ${\dot{\bar{n}}}_{{m}_{1}}$ = 300 quanta per second, which is substantial given the approximately microsecond timescale of the quantum simulation. However, this only reduced the contrast of the observed tunnelling and was not detrimental to the qualitative behaviour of the simulation. Further, the heating rate could be improved with technical upgrades to the system, meaning that this is not a limitation of the method.

#### Loop: preparing initial states

For the ${{\mathbb{Z}}}_{2}$ link, we prepared Fock states via blue sideband transitions. However, for two ions, this approach broke down because our laser addressed both qubits globally, with equal coupling strength. As a result, the blue sideband transition entangled both qubits with the oscillator, thereby preventing the preparation of well-defined Fock states.

To overcome this, we first temporarily shelved ion 1 out of the qubit manifold by transferring the state $\left\vert {\downarrow }_{{\ell }_{1}}\right\rangle$ to an auxiliary level, $\left\vert {\uparrow }_{{\rm{aux}}}\right\rangle =\left\vert {S}_{1/2},{m}_{j}=1/2\right\rangle$. This placed ion 1 in a spectrally isolated state that no longer coupled to the laser. As a result, only ion 2 interacted with the optical field, which allowed us to apply the techniques described for a single ion to prepare the desired motional Fock state. Finally, we reversed the shelving, thereby obtaining the target state $\left\vert {1}_{{m}_{1}},{\uparrow }_{{\ell }_{1}},{\uparrow }_{{\ell }_{2}},{0}_{{m}_{2}}\right\rangle$.

**Extended Data Fig. 2:**
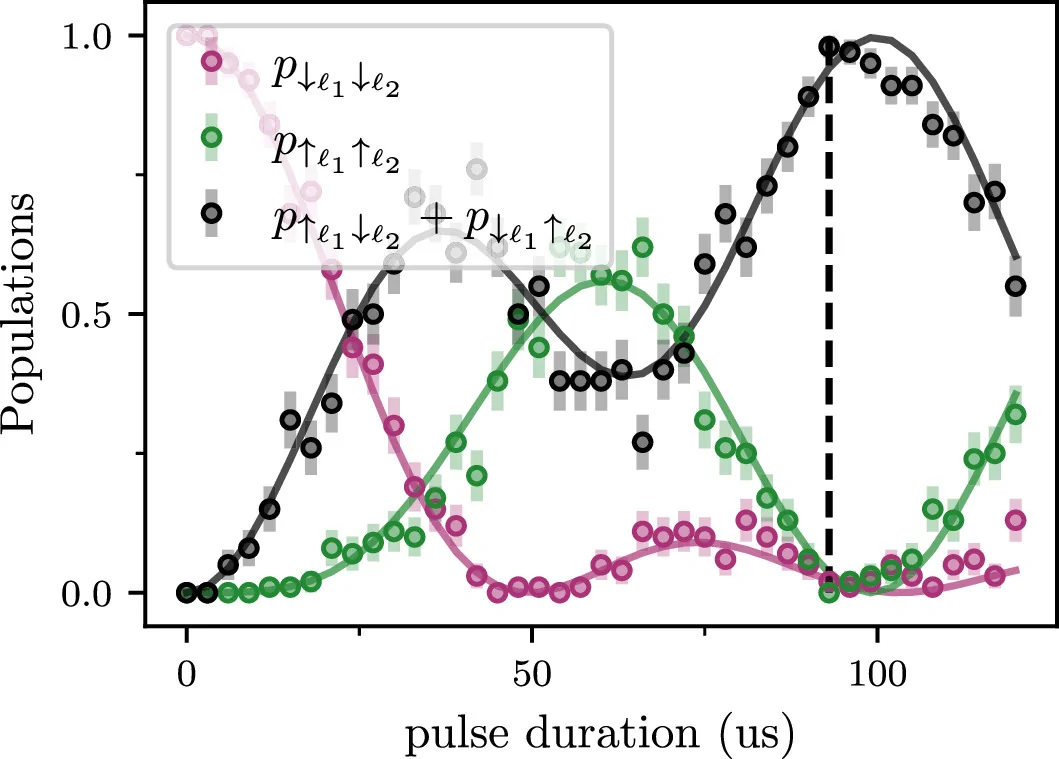
Evolution of the two ion populations as the micromotion sideband is driven for a variable duration. An asymmetric state $\left\vert {\downarrow }_{{\ell }_{1}},{\uparrow }_{{\ell }_{2}}\right\rangle$ with probability *p* = 0.98 is reached after a duration of 93*μ*s, indicated by vertical line. Solid lines are a fit to the data floating the respective Rabi frequency. Note, that despite the readout not distinguishing between $\left\vert {\downarrow }_{{\ell }_{1}},{\uparrow }_{{\ell }_{2}}\right\rangle$ and $\left\vert {\uparrow }_{{\ell }_{1}},{\downarrow }_{{\ell }_{2}}\right\rangle$ we can be certain that we prepare only one of them (convention here: $\left\vert {\downarrow }_{{\ell }_{1}},{\uparrow }_{{\ell }_{2}}\right\rangle$) but not a superposition between them as the micromotion sideband drive is not producing entanglement. We show the mean of the quantum projection measurements with sample size of 100 shots; error bars (68% confidence intervals).

Shelving was enabled by using axial micromotion sidebands that arose in our trap when ions were displaced from the radio frequency null, which naturally occurs in a two-ion crystal. By slightly shifting the axial trap position, we created an asymmetry such that ion 1 experienced a micromotion sideband coupling of the qubit transition that was twice as strong as that for ion 2. Using this asymmetry, we first prepared the qubit configuration $\left\vert {\downarrow }_{{\ell }_{1}},{\uparrow }_{{\ell }_{2}}\right\rangle$ (ref. ^57^; Extended Data Fig. 2). We then applied a radio frequency π-pulse that transferred ion 1 from $\left\vert {\downarrow }_{{\ell }_{1}}\right\rangle$ to the auxiliary shelving state $\left\vert {\uparrow }_{{\rm{aux}}}\right\rangle$, effectively isolating it from further optical interaction. Importantly, the induced micromotion imbalance did not substantially reduce the coherence time in our system.

**Extended Data Fig. 3:**
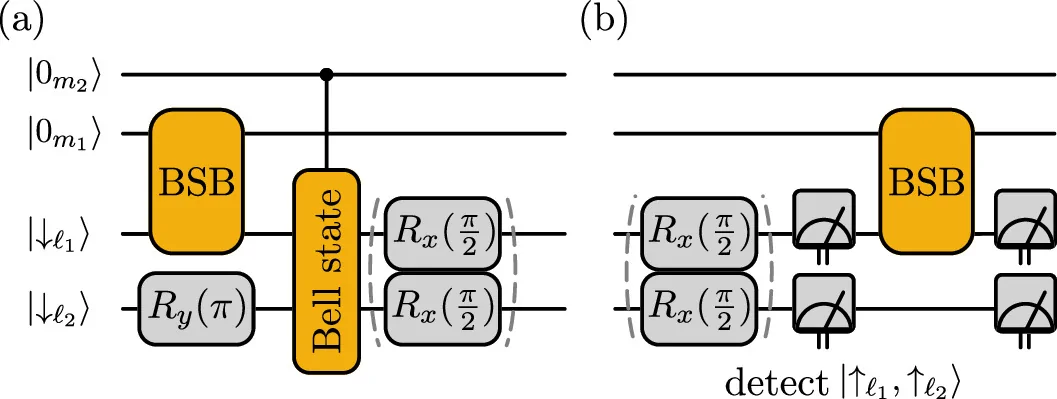
Simplified quantum circuit to prepare and read out the loop states. (**a**) The preparation sequence from the Extended Data Fig. 1(a) is followed by the Bell-state creation. We use the oscillator mode in the groundstate $\left\vert {0}_{{m}_{2}}\right\rangle$ to mediate the qubit-qubit entanglement. In parentheses is the optional *R*_*x*_(*π*/2) rotation that is needed to go from ${\Phi }_{{\ell }_{1}{\ell }_{2}}^{+}$ to ${\Psi }_{{\ell }_{1}{\ell }_{2}}^{+}$. In the real sequence, we add a midcircuit measurement just before producing the Bell-state to improve the purity of the starting state. (**b**) For the readout of the oscillator, we convert ${\Psi }_{{\ell }_{1}{\ell }_{2}}^{+}$ back to ${\Phi }_{{\ell }_{1}{\ell }_{2}}^{+}$ (*R*_*x*_(*π*/2) in parentheses) and then perform a midcircuit measurement to collapse the entangled state to $\left\vert {\uparrow }_{{\ell }_{1}},{\uparrow }_{{\ell }_{2}}\right\rangle$. The qubit measurement is analogous to the single ion case shown in Extended Data Fig. 1(d).

The quantum circuit for the state preparation sequence is shown in Extended Data Fig. 3a. We began by preparing the state $\left\vert {1}_{{m}_{1}},{\uparrow }_{{\ell }_{1}},{\uparrow }_{{\ell }_{2}},{0}_{{m}_{2}}\right\rangle$ and then applied an SDF to entangle the gauge fields. The SDF was set to be near-resonant with the motional mode encoding the second matter site, which was still near the ground state. This sequence created the Bell state $\left\vert {\Phi }_{{\ell }_{1}{\ell }_{2}}^{+}\right\rangle$ in 140 μm with fidelity ${\mathscr{ \mathcal F }}=0.943(9)$. Similarly, the state $\left\vert {\Phi }_{{\ell }_{1}{\ell }_{2}}^{-}\right\rangle$ was prepared by starting from $\left\vert {1}_{{m}_{1}},{\downarrow }_{{\ell }_{1}},{\downarrow }_{{\ell }_{2}},{0}_{{m}_{2}}\right\rangle$.

Depending on the technique used to generate the Bell state $\left\vert {\Phi }_{{\ell }_{1}{\ell }_{2}}^{+,\tilde{\phi }}\right\rangle =\frac{1}{\sqrt{2}}\left(\left\vert {\uparrow }_{{\ell }_{1}},{\uparrow }_{{\ell }_{2}}\right\rangle +{e}^{{\mathrm{i}}\tilde{\phi }}\left\vert {\downarrow }_{{\ell }_{1}},{\downarrow }_{{\ell }_{2}}\right\rangle \right)$, a relative phase $\widetilde{\phi }$ may arise. It was important to track and account for $\tilde{\phi }$ so that we could correctly apply subsequent rotations in the ${\hat{\sigma }}_{x}$–${\hat{\sigma }}_{y}$ plane or measure correlators, as discussed further in ‘Characterizing the flux and parity of Bell states’. For example, to obtain $\left\vert {1}_{{m}_{1}},{\Psi }_{{\ell }_{1}{\ell }_{2}}^{+},{0}_{{m}_{2}}\right\rangle$, we appended a global *R*_*x*_(π/2) rotation with an adjusted phase offset at the end of the preparation sequence used for $\left\vert {1}_{{m}_{1}},{\Phi }_{{\ell }_{1}{\ell }_{2}}^{+},{0}_{{m}_{2}}\right\rangle$.

#### Loop: measuring observables

Measuring the gauge field observables generalized naturally from the single-link case. By contrast, measuring the matter sites required extra care. Here we again employed a midcircuit detection scheme. This method can be directly applied to the states $\left\vert {\Phi }_{{\ell }_{1}{\ell }_{2}}^{\pm }\right\rangle$. In half of the measurement outcomes, no fluorescence was observed, indicating that the gauge fields had collapsed to state $\left\vert {\uparrow }_{{\ell }_{1}},{\uparrow }_{{\ell }_{2}}\right\rangle$, while the oscillator state remained intact.

However, for the state $\left\vert {\Psi }_{{\ell }_{1}{\ell }_{2}}^{+}\right\rangle$, a direct midcircuit measurement would always result in fluorescence from one of the qubits, thereby destroying the motional state. To avoid this, we first applied a π/2 rotation that mapped the state back to $\left\vert {\Phi }_{{\ell }_{1}{\ell }_{2}}^{+}\right\rangle$, thereby allowing the midcircuit measurement to proceed without affecting the oscillator. The full protocol is illustrated in Extended Data Fig. 3b.

### Quantifying the correlation between experimental observables

To quantify the correlations between the gauge field observables (${\bar{s}}_{\ell }^{x}$ or ${\bar{s}}_{{\ell }_{1}{\ell }_{2}}^{xx}$) and the matter excitations (${\bar{n}}_{{m}_{1}}$ and ${\bar{n}}_{{m}_{2}}$) visible in the figures, we computed Pearson’s correlation coefficient, which assumes a linear relation between the observables. We found strong correlations between the observables, as shown all three figures. The results are listed in Supplementary Table II.

### Experimental versus numerical data

#### Quantifying agreement

We quantified the match between the experimentally measured and numerically simulated observables using the root-mean-square error (RMSE):

11 $$\,\mathrm{RMSE}\,=\sqrt{\frac{1}{n}\mathop{\sum}\nolimits _{i=1}^{n}{(\,{y}_{i}^{\exp }-{y}_{i}^{\mathrm{num}})}^{2}}.$$

For the observables ${n}_{{m}_{1}}$ and ${n}_{{m}_{2}}$, we computed the RMSE directly, as their values naturally lie within the range [0, 1]. For the spin observable ${\bar{s}}_{\ell }^{x}$/${\bar{s}}_{{\ell }_{1}{\ell }_{2}}^{xx}$, we rescaled it as $({\bar{s}}_{\ell }^{x}/{\bar{s}}_{{\ell }_{1}{\ell }_{2}}^{xx}+1)/2$ to map it to the same [0, 1] range before computing the RMSE. The values for all figures are summarized in Supplementary Table III. We found good agreement, as shown in all figures.

#### Predicting the expected dynamics without symmetry-breaking noise

**Extended Data Fig. 4:**
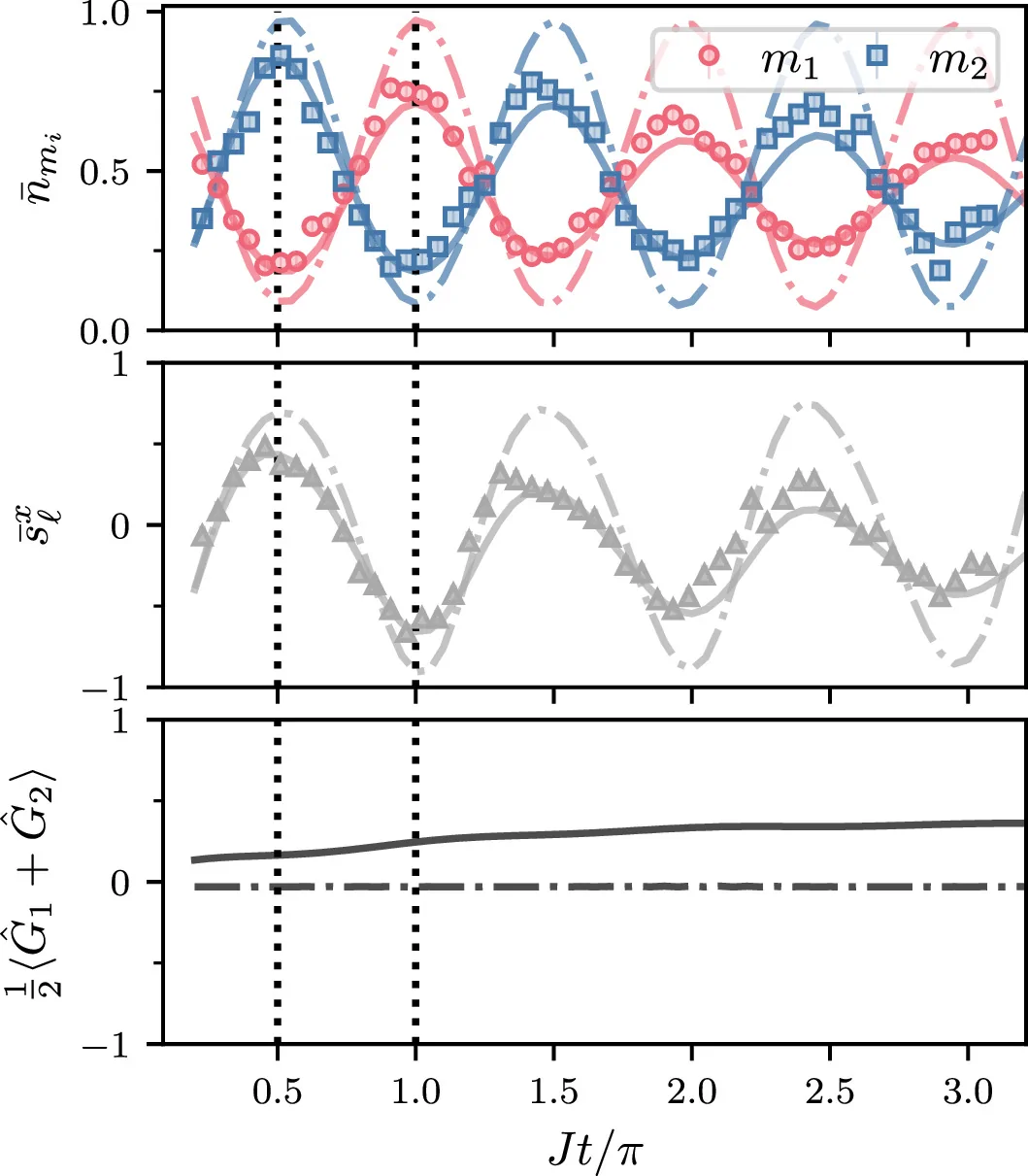
Additional numerical simulations accompanying Fig. 2. Panel three shows the numerically computed expectation value of the gauge generator $\langle {\hat{G}}_{{m}_{1}}+{\hat{G}}_{{m}_{2}}\rangle /2$. In all panels, the solid lines correspond to numerical models that include all relevant experimental error sources and are also shown in the main figure. The dash-dotted lines represent numerical results where the oscillator mode heating, the dominant source of symmetry breaking, has been artificially set to zero. This comparison highlights the impact of oscillator heating on the simulated system dynamics and its impact on gauge invariance.

We know that the leading symmetry-breaking error source in our system was due to heating of the oscillators. Justified by the good agreement between the numerical and experimental data, we numerically investigated the dynamics with a heating rate reduced to zero on both modes. Additionally, we also modelled the expectation values of the summed symmetry operators ($\langle {\hat{G}}_{{m}_{1}}+{\hat{G}}_{{m}_{2}}\rangle /2$). We show the data for this ideal case alongside the data presented in the main text in Extended Data Fig. 4.

### In-depth investigation of real-time dynamics on the ${\pmb{\mathbb{Z}}}_{\bf{2}}$ loop

#### Analytic derivation

The gauge field part of the eigenstate of the gauge-symmetry operators ${\hat{G}}_{{m}_{1}}$ and ${\hat{G}}_{{m}_{2}}$ are given by the Bell states

12 $$\begin{array}{lll}\left\vert {\Phi }_{{\ell }_{1}{\ell }_{2}}^{\pm }\right\rangle &=&\left(\left\vert {\uparrow }_{{\ell }_{1}},{\uparrow }_{{\ell }_{2}}\right\rangle \pm \left\vert {\downarrow }_{{\ell }_{1}},{\downarrow }_{{\ell }_{2}}\right\rangle\right)/\sqrt{2}\\ &=&\left(\left\vert {+}_{{\ell }_{1}},{\pm }_{{\ell }_{2}}\right\rangle +\left\vert {-}_{{\ell }_{1}},{\mp }_{{\ell }_{2}}\right\rangle \right)/\sqrt{2},\end{array}$$

13 $$\begin{array}{lll}\left\vert {\Psi }_{{\ell }_{1}{\ell }_{2}}^{\pm }\right\rangle &=&\left(\left\vert {\uparrow }_{{\ell }_{1}}{\downarrow }_{{\ell }_{2}}\right\rangle \pm \left\vert {\downarrow }_{{\ell }_{1}},{\uparrow }_{{\ell }_{2}}\right\rangle\right)/\sqrt{2}\\ &=&\left(\left\vert {\pm }_{{\ell }_{1}},{+}_{{\ell }_{2}}\right\rangle -\left\vert {\mp }_{{\ell }_{1}},{-}_{{\ell }_{2}}\right\rangle \right)/\sqrt{2}.\end{array}$$

For a single bosonic excitation in matter site 1, we then derived the following operator relations:

14 $$\begin{array}{lll}{\hat{G}}_{{m}_{1}}\left\vert {1}_{{m}_{1}},{\Phi }_{{\ell }_{1}{\ell }_{2}}^{\pm },{0}_{{m}_{2}}\right\rangle &=&(\pm 1)(-1)\left\vert {1}_{{m}_{1}},{\Phi }_{{\ell }_{1}{\ell }_{2}}^{\pm },{0}_{{m}_{2}}\right\rangle \\ &=&\mp \left\vert {1}_{{m}_{1}},{\Phi }_{{\ell }_{1}{\ell }_{2}}^{\pm },{0}_{{m}_{2}}\right\rangle ,\\ {\hat{G}}_{{m}_{2}}\left\vert {1}_{{m}_{1}},{\Phi }_{{\ell }_{1}{\ell }_{2}}^{\pm },{0}_{{m}_{2}}\right\rangle &=&(\pm 1)(1)\left\vert {1}_{{m}_{1}},{\Phi }_{{\ell }_{1}{\ell }_{2}}^{\pm },{0}_{{m}_{2}}\right\rangle \\ &=&\pm \left\vert {1}_{{m}_{1}},{\Phi }_{{\ell }_{1}{\ell }_{2}}^{\pm },{0}_{{m}_{2}}\right\rangle ,\\ {\hat{G}}_{{m}_{1}}\left\vert {1}_{{m}_{1}},{\Psi }_{{\ell }_{1}{\ell }_{2}}^{\pm },{0}_{{m}_{2}}\right\rangle &=&(\pm 1)(-1)\left\vert {1}_{{m}_{1}},{\Psi }_{{\ell }_{1}{\ell }_{2}}^{\pm },{0}_{{m}_{2}}\right\rangle \\ &=&\mp \left\vert {1}_{{m}_{1}},{\Psi }_{{\ell }_{1}{\ell }_{2}}^{\pm },{0}_{{m}_{2}}\right\rangle ,\\ {\hat{G}}_{{m}_{2}}\left\vert {1}_{{m}_{1}},{\Psi }_{{\ell }_{1}{\ell }_{2}}^{\pm },{0}_{{m}_{2}}\right\rangle &=&(\pm 1)(1)\left\vert {1}_{{m}_{1}},{\Psi }_{{\ell }_{1}{\ell }_{2}}^{\pm },{0}_{{m}_{2}}\right\rangle \\ &=&\pm \left\vert {1}_{{m}_{1}},{\Psi }_{{\ell }_{1}{\ell }_{2}}^{\pm },{0}_{{m}_{2}}\right\rangle ,\end{array}$$

and similarly for a single excitation in matter site 2. To understand the dynamics, we considered which of those eigenstates were connected through the Hamiltonian in equation (4). Let us assume first that *h* = 0. We found for the gauge field:

15 $$\begin{array}{ccc}\left\langle {\Phi }_{{\ell }_{1}{\ell }_{2}}^{\mp }|\,J\left({\hat{\sigma }}_{{\ell }_{1}}^{z}+{\hat{\sigma }}_{{\ell }_{2}}^{z}\right)| {\Phi }_{{\ell }_{1}{\ell }_{2}}^{\pm }\right\rangle & = & 2J,\\\left\langle {\Psi }_{{\ell }_{1}{\ell }_{2}}^{\mp }|\,J\left({\hat{\sigma }}_{{\ell }_{1}}^{z}+{\hat{\sigma }}_{{\ell }_{2}}^{z}\right)| {\Phi }_{{\ell }_{1}{\ell }_{2}}^{\pm }\right\rangle & = & 0,\\\left\langle {\Psi }_{{\ell }_{1}{\ell }_{2}}^{\mp }|\,J\left({\hat{\sigma }}_{{\ell }_{1}}^{z}+{\hat{\sigma }}_{{\ell }_{2}}^{z}\right)| {\Psi }_{{\ell }_{1}{\ell }_{2}}^{\pm }\right\rangle & = & 0,\\\left\langle {\Psi }_{{\ell }_{1}{\ell }_{2}}^{\pm }|\,J\left({\hat{\sigma }}_{{\ell }_{1}}^{z}+{\hat{\sigma }}_{{\ell }_{2}}^{z}\right)| {\Psi }_{{\ell }_{1}{\ell }_{2}}^{\pm }\right\rangle & = & 0.\end{array}$$

For the matter field part, we found:

16 $$\left\langle {0}_{{m}_{1}},{1}_{{m}_{2}}\left| \left({\hat{a}}_{{m}_{1}}{\hat{a}}_{{m}_{2}}^{\dagger }+{\rm{H}}.{\rm{c}}.\right)\right| {1}_{{m}_{1}},{0}_{{m}_{2}}\right\rangle =1.$$

This results in tunnelling only for states with gauge field configuration ${\Phi }_{{\ell }_{1}{\ell }_{2}}^{\pm }$. That is, $\vert {1}_{{m}_{1}},{\Phi }_{{\ell }_{1}{\ell }_{2}}^{\pm },{0}_{{m}_{2}}\rangle \leftrightarrow\vert {0}_{{m}_{1}},{\Phi }_{{\ell }_{1}{\ell }_{2}}^{\mp },{1}_{{m}_{2}}\rangle$ form a two-level system. If we assume $\vert {1}_{{m}_{1}},{\Phi }_{{\ell }_{1}{\ell }_{2}}^{+},{0}_{{m}_{2}}\rangle$ as the starting state, we found:

17 $$\left\vert \varphi (t)\right\rangle =\cos (2Jt)\left\vert {1}_{{m}_{1}},{\Phi }_{{\ell }_{1}{\ell }_{2}}^{+},{0}_{{m}_{2}}\right\rangle +{\mathrm{i}}\sin (2Jt)\left\vert {0}_{{m}_{1}},{\Phi }_{{\ell }_{1}{\ell }_{2}}^{-},{1}_{{m}_{2}}\right\rangle ,$$

which is equation (6). However, for the gauge field configuration ${\Psi }_{{\ell }_{1}{\ell }_{2}}^{\pm }$, the tunnelling is forbidden due to Aharonov–Bohm interference. If we introduce the electric field ∣*h*∣ > 0, we found the only (extra) non-vanishing term:

18 $$\left\langle {\Psi }_{{\ell }_{1}{\ell }_{2}}^{+}\left| h\left({\hat{\sigma }}_{{\ell }_{1}}^{x}+{\hat{\sigma }}_{{\ell }_{2}}^{x}\right)\right| {\Phi }_{{\ell }_{1}{\ell }_{2}}^{+}\right\rangle =2h.$$

Thus, under the full Hamiltonian, we found the three-level Λ-system dynamics $\left\vert {1}_{{m}_{1}},{\Phi }_{{\ell }_{1}{\ell }_{2}}^{-},{0}_{{m}_{2}}\right\rangle \mathop{\leftrightarrow }\limits^{2J}\left\vert {0}_{{m}_{1}},{\Phi }_{{\ell }_{1}{\ell }_{2}}^{+},{1}_{{m}_{2}}\right\rangle \mathop{\leftrightarrow }\limits^{2h}\left\vert {0}_{{m}_{1}},{\Psi }_{{\ell }_{1}{\ell }_{2}}^{+},{1}_{{m}_{2}}\right\rangle$, whereas $\left\vert {\Psi }_{{\ell }_{1}{\ell }_{2}}^{-}\right\rangle$ never participates.

#### Characterizing the flux and parity of Bell states

**Extended Data Fig. 5:**
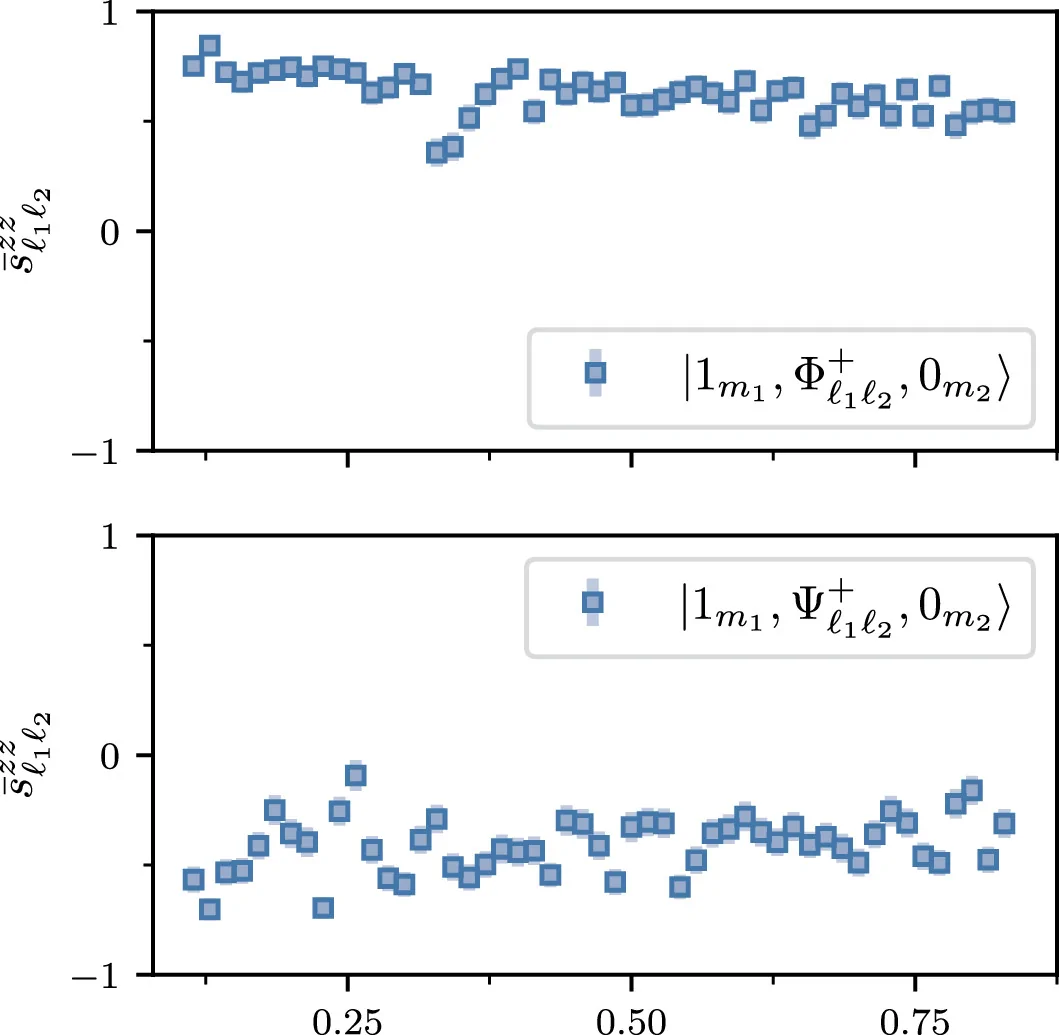
We measure the observable ${\bar{\boldsymbol{s}}}_{\pmb{\ell }_{\bf{1}}{\pmb{\ell }}_{\bf{2}}}^{\bf{zz}}={\pmb{\langle}} {\hat{\pmb{\sigma }}}_{{{\pmb\ell }}_{\bf{1}}}^{\bf{z}}{\hat{\pmb{\sigma }}}_{\pmb{\ell }_{\bf{2}}}^{\bf{z}}\pmb{\rangle}$, which is related to the flux through the loop, as a function of evolution time. The measurements are performed for the initial states $\left\vert {1}_{{m}_{1}},{\Phi }_{{\ell }_{1}{\ell }_{2}}^{+},{0}_{{m}_{2}}\right\rangle$ and $\left\vert {1}_{{m}_{1}},{\Psi }_{{\ell }_{1}{\ell }_{2}}^{+},{0}_{{m}_{2}}\right\rangle$. In the ideal case governed by the loop Hamiltonian ${\hat{H}}_{{{\mathbb{Z}}}_{2}}^{{\rm{l}}oop}$, the flux, and therefore ${\bar{s}}_{{\ell }_{1}{\ell }_{2}}^{zz}$, is expected to remain constant at 1 for $\left\vert {\Phi }_{{\ell }_{1}{\ell }_{2}}^{+}\right\rangle$ and − 1 for $\left\vert {\Psi }_{{\ell }_{1}{\ell }_{2}}^{+}\right\rangle$. In our data, $\bar{s}^{zz}_{\ell_1\ell_2}$ stays largely unchanged, consistent with this expectation. However, we observe a slow decay attributed to decoherence, and small kinks in the signal that we attribute to spectral features in the noise present in our system. We show the mean of the quantum projection measurements; error bars (68% confidence intervals) are omitted when smaller than the markers. Post-selection during the conditional readout results in ≥203 shots per point, depending on the data point.

Aside from measuring ${\bar{s}}_{{\ell }_{1}{\ell }_{1}}^{xx}$ as shown in Fig. 4, we also measured ${\bar{s}}_{{\ell }_{1}{\ell }_{2}}^{zz}=\langle {\hat{\sigma }}_{{\ell }_{1}}^{z}{\hat{\sigma }}_{{\ell }_{2}}^{z}\rangle$, which is proportional to the flux through the loop. We show the measurement results in Extended Data Fig. 5. The observable stayed constant, apart from decoherence-induced decay.

Further, to understand the gauge field dynamics precisely for the initial state $\left\vert {1}_{{m}_{1}},{\Phi }_{{\ell }_{1}{\ell }_{2}}^{+},{0}_{{m}_{2}}\right\rangle$, we expanded the analytic expression by considering equation (6) and tracing out the motion:

19 $$\begin{array}{lll}\rho &=&{\cos }^{2}(2Jt)\left\vert {\Phi }_{{\ell }_{1}{\ell }_{2}}^{+}\right\rangle \left\langle {\Phi }_{{\ell }_{1}{\ell }_{2}}^{+}\right\vert +{\sin }^{2}(2Jt)\left\vert {\Phi }_{{\ell }_{1}{\ell }_{2}}^{-}\right\rangle \left\langle {\Phi }_{{\ell }_{1}{\ell }_{2}}^{-}\right\vert \\ &=&\frac{1}{2}\left(\left\vert {\uparrow }_{{\ell }_{1}}{\uparrow }_{{\ell }_{2}}\right\rangle \left\langle {\uparrow }_{{\ell }_{1}}{\uparrow }_{{\ell }_{2}}\right\vert +\left\vert {\downarrow }_{{\ell }_{1}}{\downarrow }_{{\ell }_{2}}\right\rangle \left\langle {\downarrow }_{{\ell }_{1}}{\downarrow }_{{\ell }_{2}}\right\vert\right)\\ &&+\frac{\cos (4Jt)}{2}\left(\left\vert {\uparrow }_{{\ell }_{1}}{\uparrow }_{{\ell }_{2}}\right\rangle \left\langle {\downarrow }_{{\ell }_{1}}{\downarrow }_{{\ell }_{2}}\right\vert +\left\vert {\downarrow }_{{\ell }_{1}}{\downarrow }_{{\ell }_{2}}\right\rangle \left\langle {\uparrow }_{{\ell }_{1}}{\uparrow }_{{\ell }_{2}}\right\vert\right).\end{array}$$

**Extended Data Fig. 6:**
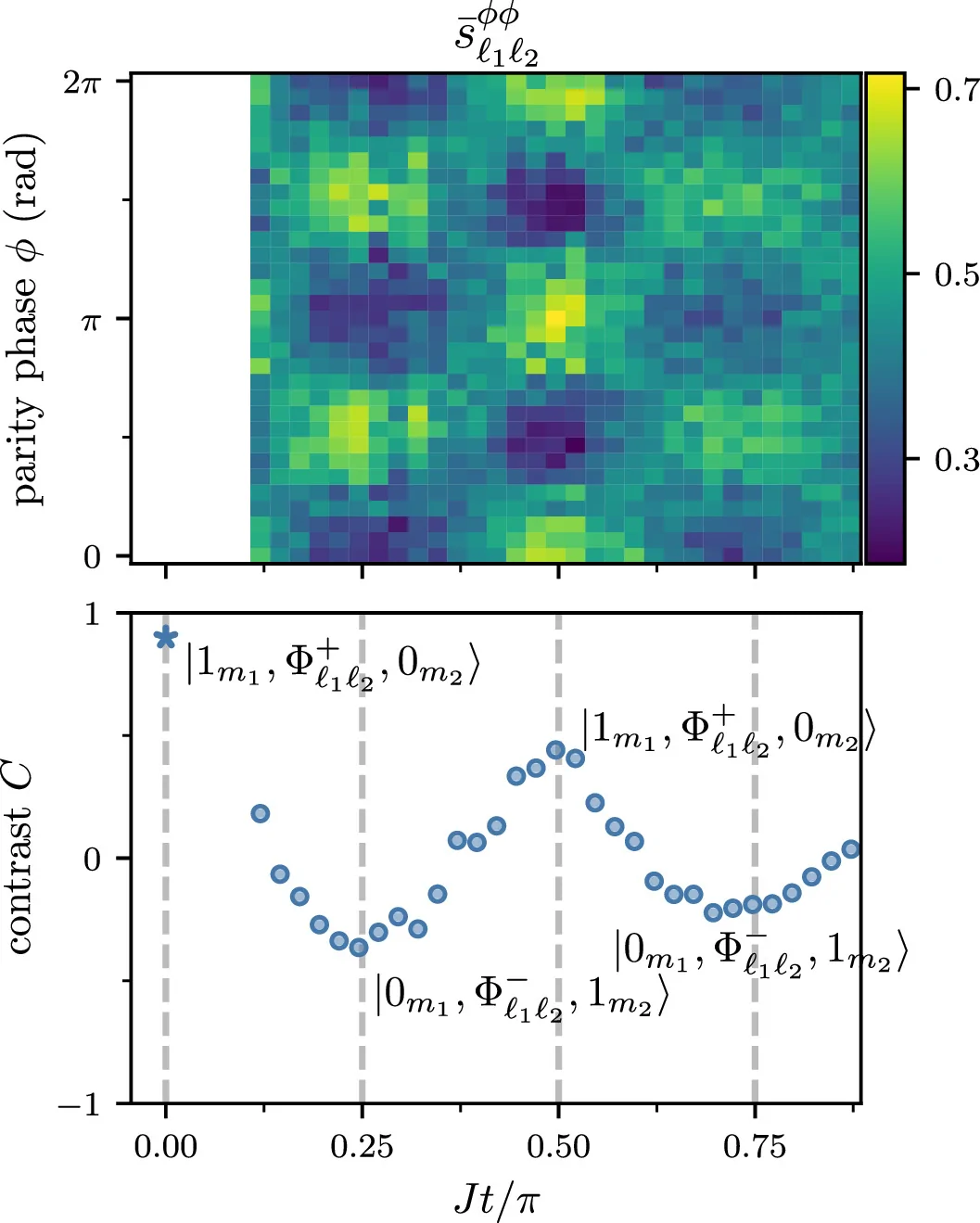
Investigating the expectation value ${\bar{\boldsymbol{s}}}_{\pmb{\ell }_{\bf{1}}{\pmb{\ell }}_{\bf{2}}}^{\pmb{\phi \phi }}={\pmb\langle }{\hat{\pmb{\sigma }}}_{\pmb{\ell }_{\bf{1}}}^{\pmb{\phi }}{\hat{\pmb{\sigma }}}_{\pmb{\ell }_{\bf{2}}}^{\pmb{\phi }}\pmb{\rangle}$ as a function of the applied tunnelling duration. In the heatmap, we show the raw data. In the lower plot, we display the extracted contrast found by fitting a sinusoid at every duration. Errors (68% confidence interval) are inferred from the estimated covariance matrix of the fit and are smaller than the marker size. We have indicated, where the measured contrast *C* is closest to the maximally entangled states of the system. The minimal duration *Jt*/*π* = 0.24 is given by the pulse ramping duration. Additionally, in the lower panel, we added the contrast of the initially prepared Bell state $\left\vert {\Phi }_{{\ell }_{1}{\ell }_{2}}^{+}\right\rangle$ (star).

At 4*Jt* = π/2, we found the completely disentangled state $\rho =(\vert {\uparrow }_{{\ell }_{1}}{\uparrow }_{{\ell }_{2}}\rangle\langle {\uparrow }_{{\ell }_{1}}{\uparrow }_{{\ell }_{2}}\vert +\vert {\downarrow }_{{\ell }_{1}}{\downarrow }_{{\ell }_{2}}\rangle\langle {\downarrow }_{{\ell }_{1}}{\downarrow }_{{\ell }_{2}}\vert )/2$. We extracted the contrast C=cos(4Jt) as a function of the duration of the tunnelling. To extract the contrast, we measured ${\bar{s}}_{{\ell }_{1}{\ell }_{2}}^{\phi \phi }=\langle \cos (\phi )({\hat{\sigma }}_{{\ell }_{1}}^{x}{\hat{\sigma }}_{{\ell }_{2}}^{x})+\sin (\phi )({\hat{\sigma }}_{{\ell }_{1}}^{y}{\hat{\sigma }}_{{\ell }_{2}}^{y})\rangle$ and varied *ϕ* (Extended Data Fig. 6). This is also known as a parity measurement.

As mentioned previously, depending on the technique used to produce the Bell state $\vert {\Phi }_{{\ell }_{1}{\ell }_{2}}^{+,\tilde{\phi }}\rangle =\frac{1}{\sqrt{2}}(\vert {\uparrow }_{{\ell }_{1}},{\uparrow }_{{\ell }_{2}}\rangle +{e}^{{\mathrm{i}}\tilde{\phi }}\vert {\downarrow }_{{\ell }_{1}},{\downarrow }_{{\ell }_{2}}\rangle)$, a relative phase $\tilde{\phi }$ may arise, which would shift the parity fringe shown in Extended Data Fig. 6. It is important to track and account for $\tilde{\phi }$ to correctly measure the ${\bar{s}}_{{\ell }_{1}{\ell }_{2}}^{xx}$ correlation, which effectively corresponds to measuring a rotated correlation ${\bar{s}}_{{\ell }_{1}{\ell }_{2}}^{\tilde{\phi }\tilde{\phi }}$.

### Proof of $[{\hat{H}}_{{{\mathbb{Z}}}_{2}}^{{\rm{link}}},{\hat{G}}_{{m}_{i}}]=0$

The electric field part $[h{\hat{\sigma }}_{\ell }^{z},{\hat{G}}_{{m}_{i}}]=0$ can be seen directly. What is left is to prove that $[{\hat{\sigma }}_{\ell }^{z}({\hat{a}}_{{m}_{1}}{\hat{a}}_{{m}_{2}}^{\dagger }+{\hat{a}}_{{m}_{1}}^{\dagger }{\hat{a}}_{{m}_{2}}),{\hat{G}}_{{m}_{i}}]=0$. First, let us calculate

20 $$\begin{array}{lll}{({{\rm{e}}}^{{\rm{i}}\pi {\hat{a}}^{\dagger }\hat{a}})}^{\dagger }{\hat{a}}^{\dagger }({{\rm{e}}}^{{\rm{i}}\pi {\hat{a}}^{\dagger }\hat{a}})\left\vert n\right\rangle &=&{({{\rm{e}}}^{{\rm{i}}\pi {\hat{a}}^{\dagger }\hat{a}})}^{\dagger }{\hat{a}}^{\dagger }{(-1)}^{n}\left\vert n\right\rangle \\ &=&{({{\rm{e}}}^{{\rm{i}}\pi {\hat{a}}^{\dagger }\hat{a}})}^{\dagger }{(-1)}^{n}\sqrt{n+1}\left\vert n+1\right\rangle \\ &=&{(-1)}^{n+1}{(-1)}^{n}\sqrt{n+1}\left\vert n+1\right\rangle \\ &=&-{\hat{a}}^{\dagger }\left\vert n\right\rangle ,\end{array}$$

and similarly

21 $${({e}^{{\mathrm{i}}\pi {\hat{a}}^{\dagger }\hat{a}})}^{\dagger }\hat{a}({e}^{{\mathrm{i}}\pi {\hat{a}}^{\dagger }\hat{a}})\left\vert n\right\rangle =-\hat{a}\left\vert n\right\rangle.$$

Thus, we can make use of the identities

22 $${\hat{\sigma }}_{\ell }^{x}{\hat{\sigma }}_{\ell }^{z}{\hat{\sigma }}_{\ell }^{x}=-{\hat{\sigma }}_{\ell }^{z},$$

23 $${\left({{\rm{e}}}^{{\rm{i}}\pi {\hat{a}}_{{m}_{1}}^{\dagger }{\hat{a}}_{{m}_{1}}}\right)}^{\dagger }\left({\hat{a}}_{{m}_{1}}{\hat{a}}_{{m}_{2}}^{\dagger }+{\hat{a}}_{{m}_{1}}^{\dagger }{\hat{a}}_{{m}_{2}}\right){{\rm{e}}}^{{\rm{i}}\pi {\hat{a}}_{{m}_{1}}^{\dagger }{\hat{a}}_{{m}_{1}}}=-\left({\hat{a}}_{{m}_{1}}{\hat{a}}_{{m}_{2}}^{\dagger }+{\hat{a}}_{{m}_{1}}^{\dagger }{\hat{a}}_{{m}_{2}}\right),$$

to prove that for the generator ${\hat{G}}_{{m}_{1}}$

24 $$\underbrace{{({\hat{\sigma }}_{\ell }^{x}{{\rm{e}}}^{{\rm{i}}\pi {\hat{a}}_{{m}_{1}}^{\dagger }{\hat{a}}_{{m}_{1}}})}^{\dagger }}_{{\hat{G}}_{{m}_{1}}^{\dagger }}{\hat{H}}_{{{\mathbb{Z}}}_{2}}^{{\rm{link}}}\underbrace{({\hat{\sigma }}_{\ell }^{x}{{\rm{e}}}^{{\rm{i}}\pi {\hat{a}}_{{m}_{1}}^{\dagger }{\hat{a}}_{{m}_{1}}})}_{{\hat{G}}_{{m}_{1}}}=$$

25 $$={{\rm{e}}}^{-{\rm{i}}\pi {\hat{a}}_{{m}_{1}}^{\dagger }{\hat{a}}_{{m}_{1}}}{\hat{\sigma }}_{\ell }^{x}{\hat{H}}_{{{\mathbb{Z}}}_{2}}^{{\rm{link}}}{\hat{\sigma }}_{\ell }^{x}{{\rm{e}}}^{{\rm{i}}\pi {\hat{a}}_{{m}_{1}}^{\dagger }{\hat{a}}_{{m}_{1}}}$$

26 $$\mathop{=}\limits^{(22)}-{{\rm{e}}}^{-{\rm{i}}\pi {\hat{a}}_{{m}_{1}}^{\dagger }{\hat{a}}_{{m}_{1}}}{\hat{H}}_{{{{{\mathbb{Z}}}}}_{2}}^{\mathrm{link}}{{\rm{e}}}^{{\rm{i}}\pi {\hat{a}}_{{m}_{1}}^{\dagger }{\hat{a}}_{{m}_{1}}}$$

27 $$\mathop{=}\limits^{(23)}{\hat{H}}_{{{{{\mathbb{Z}}}}}_{2}}^{\mathrm{link}},$$

and the same for ${\hat{G}}_{{m}_{2}}$. This extends to $[{\hat{H}}_{{{\mathbb{Z}}}_{2}}^{{\rm{loop}}},{\hat{G}}_{{m}_{i}}]=0$ as well.

### Further investigations and future work

#### Competition between the electric field, tunnelling and the Aharonov–Bohm effect

**Extended Data Fig. 7:**
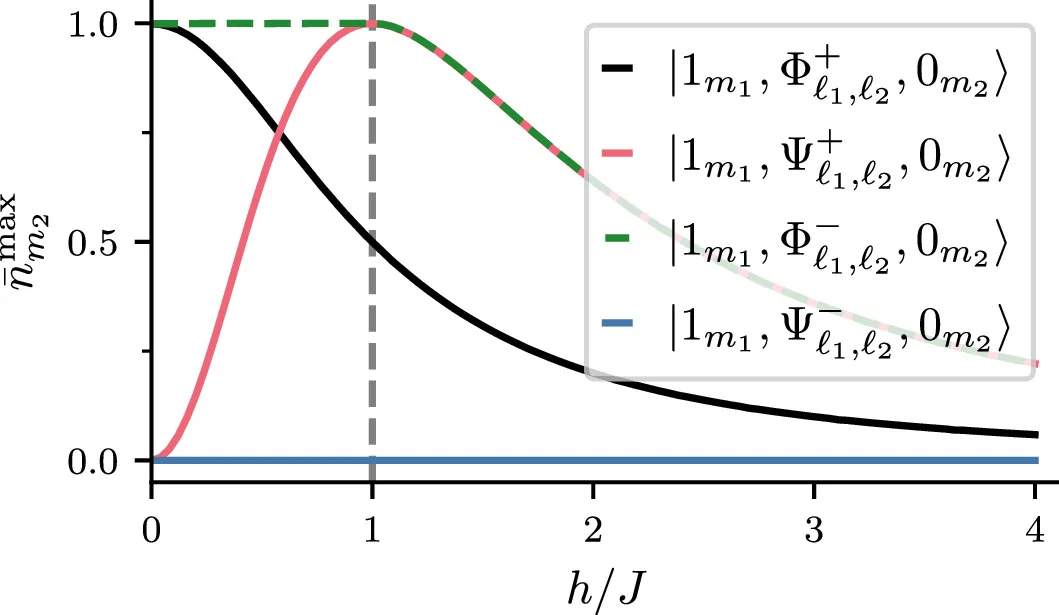
Numerical simulation of maximal tunnelling magnitude ${\bar{\boldsymbol{n}}}_{\boldsymbol{m}_{\bf{2}}}^{\max }=\pmb{\max} (\pmb{\langle }{\boldsymbol{n}}_{\boldsymbol{m}_{\bf{2}}}\pmb\rangle )$ for different ratios of *h*/*J* for the four possible initial entangled gauge field configurations of the loop. In the limit of *h* → *∞* the tunnelling magnitude vanishes for all the starting states. For *h* = 0, the tunnelling magnitude is maximised for the Bell-states ${\Phi }_{{\ell }_{1}{\ell }_{2}}^{\pm }$ and completely suppressed for ${\Psi }_{{\ell }_{1}{\ell }_{2}}^{\pm }$ as a result of the AB-interference. For ${\Psi }_{{\ell }_{1}{\ell }_{2}}^{+}$, however, any finite electric field perturbs the perfect destructive AB-interference, and tunnelling occurs.

As for a single ion, there is inherent competition between the electric field term and the tunnelling term. However, there is another dynamic on the loop due to the Aharonov–Bohm interference. These three effects now all compete, leading to non-trivial dynamics. We briefly investigated the full Λ-system dynamics $\left\vert {1}_{{m}_{1}},{\Phi }_{{\ell }_{1}{\ell }_{2}}^{-},{0}_{{m}_{2}}\right\rangle \mathop{\leftrightarrow }\limits^{2J}\left\vert {0}_{{m}_{1}},{\Phi }_{{\ell }_{1}{\ell }_{2}}^{+},{1}_{{m}_{2}}\right\rangle \mathop{\leftrightarrow }\limits^{2h}\left\vert {0}_{{m}_{1}},{\Psi }_{{\ell }_{1}{\ell }_{2}}^{+},{1}_{{m}_{2}}\right\rangle$ and the isolated $\left\vert {\varPsi }_{{\ell }_{1}{\ell }_{2}}^{-}\right\rangle$ state using numerical simulations. The effect of a non-vanishing electric field on the dynamics is shown in Extended Data Fig. 7.

We calculated the maximum tunnelling amplitude ${\bar{n}}_{{m}_{2},\max }$ for different ratios of *h*/*J* considering starting states $\left\vert {1}_{{m}_{1}},{\Phi }_{{\ell }_{1}{\ell }_{2}}^{\pm },{0}_{{m}_{2}}\right\rangle$ and $\left\vert {1}_{{m}_{1}},{\Psi }_{{\ell }_{1}{\ell }_{2}}^{\pm },{0}_{{m}_{2}}\right\rangle$. For $\left\vert {1}_{{m}_{1}},{\Phi }_{{\ell }_{1}{\ell }_{2}}^{+},{0}_{{m}_{2}}\right\rangle$, where both gauge fields are aligned, we found the same dynamics as on a single gauge link; the tunnelling magnitude gradually became suppressed as *h* → *∞*. For $\left\vert {1}_{{m}_{1}},{\Phi }_{{\ell }_{1}{\ell }_{2}}^{-},{0}_{{m}_{2}}\right\rangle$ the tunnelling magnitude was a maximum at *h* = 0 and stayed a maximum until *h* > *J*, after which it was gradually suppressed as *h* → *∞*. However, when the gauge fields were anti-aligned ($\left\vert {\Psi }_{{\ell }_{1}{\ell }_{2}}^{\pm }\right\rangle$) and, hence, *ϕ*_AB_ = π, the tunnelling was fully suppressed for *h* = 0 and for *h* → *∞*. For $\vert {\Psi }_{{\ell }_{1}{\ell }_{2}}^{-}\rangle$, the destructive interference was perfect for any *h*. However, for $\vert {\varPsi }_{{\ell }_{1}{\ell }_{2}}^{+}\rangle$, there was an intermediate regime (centred at *J* = *h*) where tunnelling was possible. Here, the presence of the electric field term destroyed the perfect destructive interference due to the Aharonov–Bohm effect.

#### Even and odd parity squeezed matter

**Extended Data Fig. 8:**
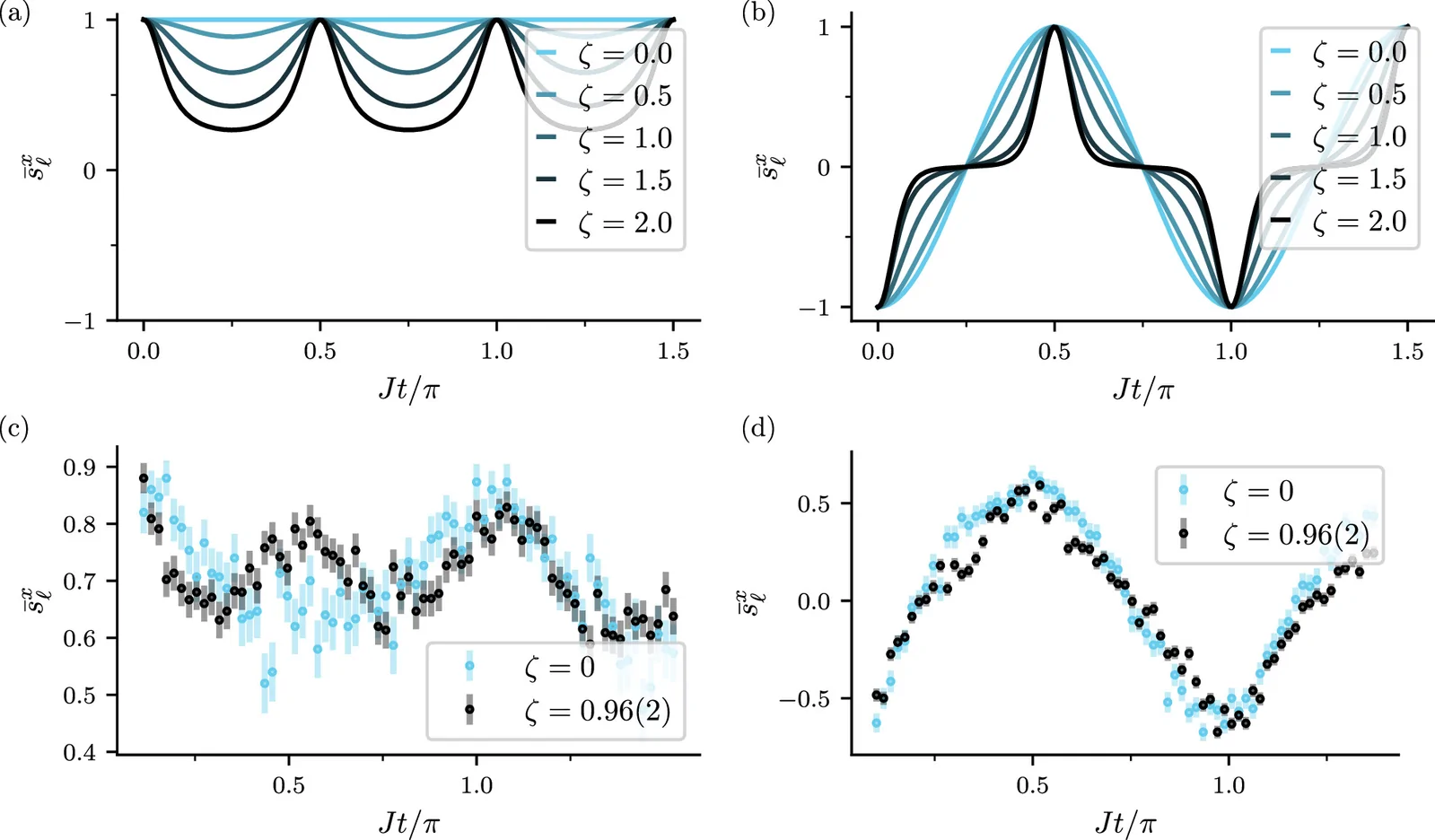
Expectation value of ${\bar{\boldsymbol{s}}}_{\pmb\ell }^{\boldsymbol{x}}$ as a function of tunnelling duration. (**a**) Starting from the state $\left\vert {\zeta }_{{m}_{1}}^{+},{+}_{\ell },{0}_{{m}_{2}}\right\rangle$, representing no matter excitation at either site, that is, ${q}_{{m}_{1}}={q}_{{m}_{2}}=0$, with variable squeezing *ζ* applied to $\left\vert {0}_{{m}_{1}}\right\rangle$. The resulting correlation changes from no tunnelling at all to a pattern of partial tunnelling with full revival at each swap duration. Corresponding experimental data are shown in panel (**c**). (**b**) Expectation value of ${\bar{s}}_{\ell }^{x}$ as a function of tunnelling duration, starting from the state $\left\vert {\zeta }_{{m}_{1}}^{-},{-}_{\ell },{0}_{{m}_{2}}\right\rangle$, representing a matter excitation at *m*_1_, that is, ${q}_{{m}_{1}}=1$, ${q}_{{m}_{2}}=0$, with variable squeezing *ζ* applied to $\left\vert {1}_{{m}_{1}}\right\rangle$. The resulting correlation changes from sinusoidal to an increasingly sharp feature which, in the (nonphysical) limit *ζ* → *∞*, becomes a delta function at the full interchange times. Corresponding experimental data are shown in panel (**d**). (**c**) Experimentally measured expectation value of ${\bar{s}}_{\ell }^{x}$ as a function of tunnelling duration, starting from the state $\left\vert {\zeta }_{{m}_{1}}^{+},{+}_{\ell },{0}_{{m}_{2}}\right\rangle$. For *ζ* = 0 (blue points), periodic spin correlation with *Jt*/*π* = 1 is observed, corresponding to regular tunnelling of thermal states near $\left\vert {0}_{{m}_{i}}\right\rangle$. For ∣*ζ*∣ = 0.96(2) (black points), a partial revival appears at *Jt*/*π* = 0.5, as predicted by simulations. For easier comparison, the baseline of the squeezed dynamics was adjusted. (**d**) Experimentally measured expectation value of ${\bar{s}}_{\ell }^{x}$ as a function of tunnelling duration, starting from the state $\left\vert {\zeta }_{{m}_{1}}^{-},{-}_{\ell },{0}_{{m}_{2}}\right\rangle$. For *ζ* = 0 (blue points), spin periodicity with *Jt*/*π* = 1 is observed. For ∣*ζ*∣ = 0.96(2) (black points), the feature sharpens slightly, though less pronounced than predicted. To highlight the deviation from a sinusoidal pattern, the contrast of the squeezed dynamics was scaled by a factor of 1.45. The reduced contrast suggests decoherence affects the dynamics. Points in panels (**c**,**d**) show mean values from quantum projection measurements with 100 shots for the unsqueezed data and 300 shots for the squeezed data; error bars denote 68% confidence intervals.

A strength of our mapping is that the matter sites are true bosons. As such, they can support more than a single excitation. For the ${{\mathbb{Z}}}_{2}$ LGT, we are still interested in states that have a well-defined charge and, hence, are eigenstates of ${\hat{P}}_{{m}_{i}}\left\vert {\zeta }_{{m}_{i}}^{\pm }\right\rangle \equiv \exp ({\rm{i}}\pi {\hat{a}}_{{m}_{i}}^{\dagger }{\hat{a}}_{{m}_{i}})\left\vert {\zeta }_{{m}_{i}}^{\pm }\right\rangle =\pm \left\vert {\zeta }_{{m}_{i}}^{\pm }\right\rangle$. A particular subset of such states are the squeezed vacuum and squeezed phonon-added vacuum states^58^, namely

28 $$\begin{array}{lll}\left\vert {\zeta }_{{m}_{i}}^{+}\right\rangle &=&\hat{S}(\zeta )\left\vert {0}_{{m}_{i}}\right\rangle \\ &=&\frac{1}{\sqrt{\cosh (r)}}\mathop{\sum }\limits_{n = 0}^{\infty }\frac{\sqrt{(2n)!}}{{2}^{n}n!}{({{\rm{e}}}^{{\rm{i}}\theta }\tanh (r))}^{n}\left\vert 2{n}_{{m}_{i}}\right\rangle ,\end{array}$$

29 $$\begin{array}{lll}\left\vert {\zeta }_{{m}_{i}}^{-}\right\rangle &=&\hat{S}(\zeta )\left\vert {1}_{{m}_{i}}\right\rangle \\ &=&\frac{1}{{(\cosh (r))}^{3/2}}\mathop{\sum }\limits_{n = 0}^{\infty }\frac{\sqrt{(2n+1)!}}{{2}^{n}n!}{({{\rm{e}}}^{{\rm{i}}\theta }\tanh (r))}^{n}\left\vert 2n+{1}_{{m}_{i}}\right\rangle ,\end{array}$$

where the squeezing operator is defined as

30 $$\hat{S}(\zeta )=\exp \left(\frac{1}{2}\left({\zeta }^{* }{\hat{a}}_{{m}_{i}}^{2}-\zeta {({\hat{a}}_{{m}_{i}}^{\dagger })}^{2}\right)\right),$$

with squeezing parameter *ζ*. These states are eigenstates of the gauge-symmetry operator and can, thus, be associated with dynamical charges ${q}_{{m}_{i}}=0$ and ${q}_{{m}_{i}}=1$, respectively. As in the single-excitation case, it is of interest to study the expectation values of the observables ${\bar{n}}_{{m}_{1}}$, ${\bar{n}}_{{m}_{2}}$ and ${\bar{s}}_{\ell }^{x}$. In particular, we consider the initial state $\left\vert {\zeta }_{{m}_{1}}^{\pm },{\pm }_{\ell },{0}_{{m}_{2}}\right\rangle$ and study the tunnelling (no transverse field term). Although the mean phonon numbers ${\bar{n}}_{{m}_{1}}$ and ${\bar{n}}_{{m}_{2}}$ oscillated back and forth sinusoidally between the two sites, the dynamics of the qubit correlation changed as a function of ∣*ζ*∣ (Extended Data Fig. 8a,b).

**Extended Data Fig. 9:**
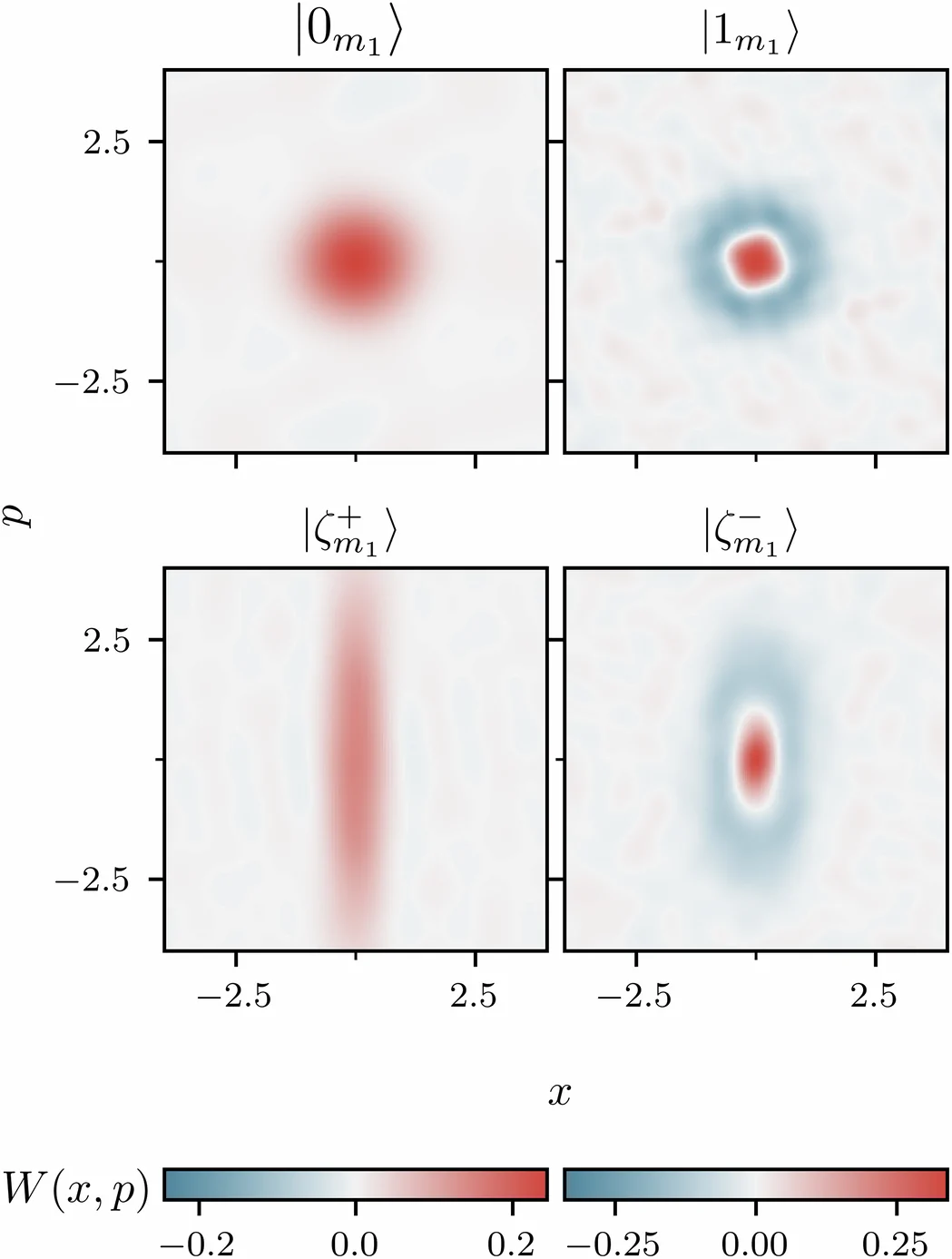
Reconstructed Wigner function for a matter site in state $\left \vert{\bf{0}}_{\boldsymbol{m}_{\bf{1}}}\right\rangle$, $\left\vert {\bf{1}}_{\boldsymbol{m}_{\bf{1}}}\right\rangle$, $\hat{\boldsymbol{S}}(\pmb\zeta )\left\vert {\bf{0}}_{\boldsymbol{m}_{\bf{1}}}\right\rangle =\left\vert {\pmb\zeta }_{\boldsymbol{m}_{\bf{1}}}^{+}\right\rangle$ with *ζ* ≈ 1.1, and $\hat{\boldsymbol{S}}(\pmb\zeta )\left\vert \bf{1}\right\rangle =\left\vert {\pmb\zeta }_{\boldsymbol{m}_{\bf{1}}}^{\bf{-}}\right\rangle$ with *ζ* = 0.96(2). The reconstruction of $\left\vert {1}_{{m}_{1}}\right\rangle$ suffers from undersampling. As a result, the shape is distorted by the sampling grid pattern (square).

**Extended Data Fig. 10:**
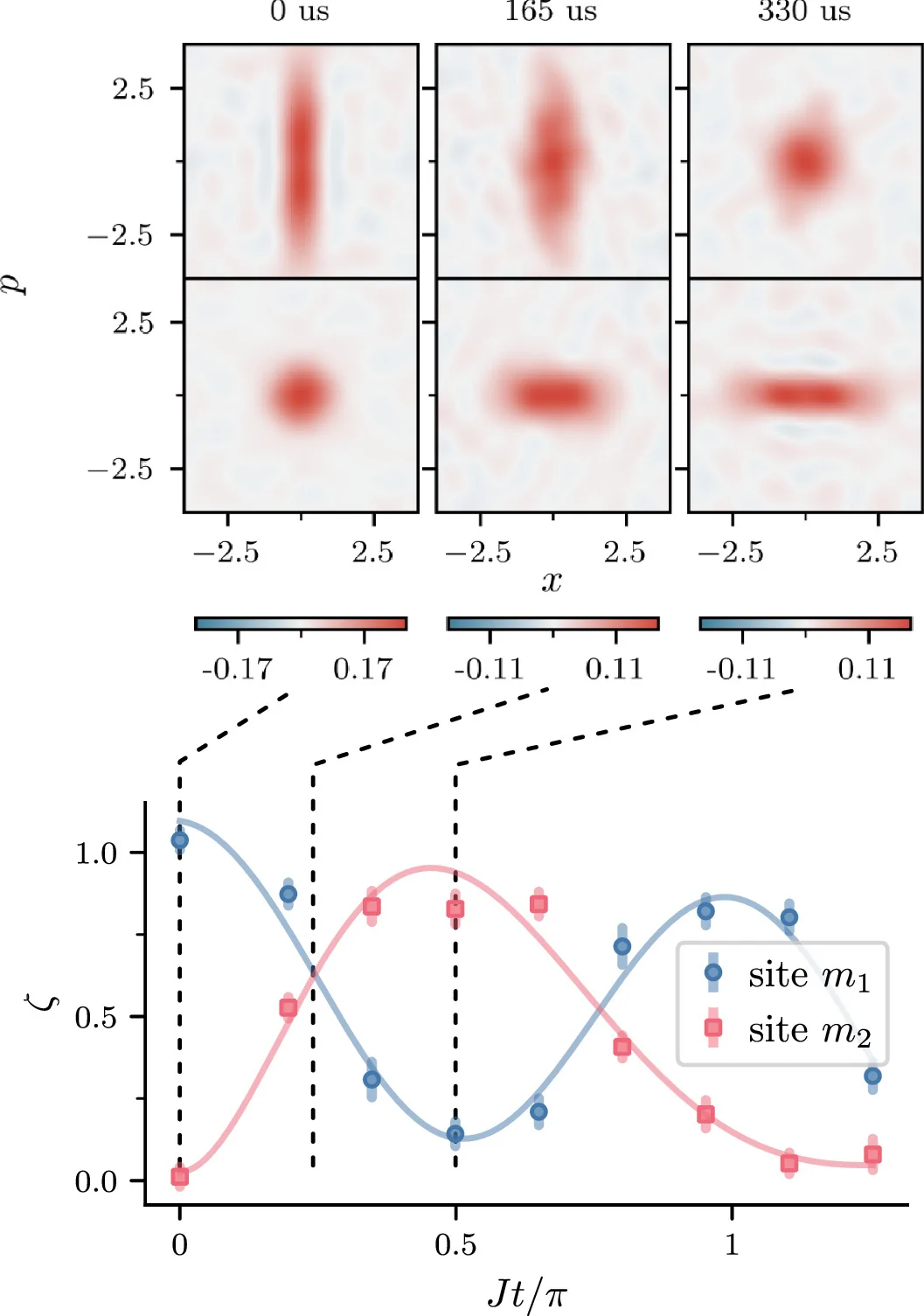
Observed tunnelling from the initial state $\left\vert {\pmb\zeta }_{\boldsymbol{m}_{\bf{1}}}^{+},{\downarrow }_{\pmb\ell },{\bf{0}}_{\boldsymbol{m}_{\bf{2}}}\right\rangle$. Extracted squeezing magnitude *ζ* at each matter site *m*_*i*_ as a function of tunnelling duration using the method of ref. ^59^; markers indicate the fitted value of *ζ*, while error bars denote 68% confidence intervals obtained from the covariance matrix of the fit. We also show decaying sinusoidal fits (solid lines). At selected durations, we additionally perform full Wigner function reconstructions using the technique of ref. ^48^.

We first verified that we could synthesize these states experimentally. Extended Data Fig. 9 displays the reconstructed Wigner function of $\left\vert {\zeta }_{{m}_{1}}^{\pm }\right\rangle$. We then verified that the squeezed matter tunnelled as we expected. We prepared a state $\left\vert {\zeta }_{{m}_{1}}^{+},{\downarrow }_{\ell },{0}_{{m}_{2}}\right\rangle$ that was initially squeezed on matter site 1 while prepared in its vacuum state on matter site *m*_2_. We measured the squeezing magnitude ∣*ζ*∣ (using the method described in refs. ^53,54,59^) on each matter site as a function of the applied tunnelling duration (Extended Data Fig. 10). We found that as matter site *m*_1_, which was initially squeezed, gradually became less squeezed, the degree of squeezing increased on matter site *m*_2_. Note, that the intermediate states at site *m*_1_ were a superposition between the squeezed state and the vacuum state ($a\left\vert r\right\rangle +b\left\vert 0\right\rangle$). Only for *a* = 0 or *b* = 0 was this state a true squeezed state with a well-defined squeezing magnitude. Nonetheless, we used it as a proxy to quantify the changing state. Subsequently, we reconstructed the full Wigner function of each mode at different instances of the tunnelling duration using the technique described in ref. ^48^.

We proceeded to measure the gauge field correlation of the tunnelling for the initial configurations $\left\vert {\zeta }_{{m}_{1}}^{+},{+}_{\ell },{0}_{{m}_{2}}\right\rangle$ (Extended Data Fig. 8c) and $\left\vert {\zeta }_{{m}_{1}}^{-},{-}_{\ell },{0}_{{m}_{2}}\right\rangle$ (Extended Data Fig. 8d). Although we did find that the pattern of the gauge field correlation resembled the simulated prediction, it was substantially less pronounced. We suspect that this was a result of the finite motional and spin coherence times in our system. Numerically investigating this problem is surprisingly challenging. In particular, we found that we needed to choose a Fock state truncation of at least 25 to faithfully represent the squeezed state. This gave a resulting hybrid state size of 2 × 25 × 25 = 1,250, which can rapidly become challenging to simulate, particularly when we considered larger lattices.

## Online content

Any methods, additional references, Nature Portfolio reporting summaries, source data, extended data, supplementary information, acknowledgements, peer review information; details of author contributions and competing interests; and statements of data and code availability are available at https://doi.org/10.1038/s41567-026-03400-6.

## Supplementary information

Supplementary Information Supplementary Tables I–III.

Peer Review File

## Source data

Source Data Fig. 2 Data for reproducing Fig 2.

Source Data Fig. 3 Data for reproducing Fig 3.

Source Data Fig. 4 Data for reproducing Fig 4.

## Data Availability

Data that can be used to reproduce to reproduce Figs. 2–4 are available via Figshare at https://doi.org/10.6084/m9.figshare.32231322 (ref. ^60^). All other data that support the plots within this paper and other findings of this study are available from the corresponding author upon reasonable request. Source data are provided with this paper.
