# Supplementary material for: Aharonov–Bohm interference in a ℤ2 lattice gauge theory on a hybrid qubit–oscillator quantum computer
Source: Nat Phys. 2026 Sep 25;22(10):1565–71. doi: 10.1038/s41567-026-03400-6 (PMC13658009; doi:10.1038/s41567-026-03400-6)
Supplement: Supplementary file 1 [file 41567_2026_3400_MOESM1_ESM.pdf]

nature physics

Supplementary information https://doi.org/10.1038/s41567-026-03400-6

Aharonov–Bohm interference in a $\mathbb{Z}_2$ lattice gauge theory on a hybrid qubit–oscillator quantum computer

In the format provided by the authors and unedited

Supplemental Material for: Observing Aharonov-Bohm Interference in a $\mathbb{Z}_2$ Lattice Gauge Theory on a Hybrid Qubit-Oscillator Quantum Computer

Table I: Experimental parameters for the real-time dynamics. All interactions embedded in a spin echo sequence have the duration t given as 2 x t_{arm} where t_{arm} is the full-width half maximum duration for which the interaction is applied in each arm. We show the used power P at the ion per SDF, the strength of the SDF $\Omega_{\alpha,\alpha'}$, detuning Δ of the SDFs used to generate the interaction, the spin-conditioning $\hat{\sigma}_\beta$ and the motional modes (ax-ip: axial in-phase, lr-ip: lower radial in-phase, ur-ip: upper radial in-phase mode of the ion crystal) used to encode the matter sites.

Fig.	P (mW)	Ω_α, $\Omega_{\alpha'}$ (2π kHz)	$\Delta/2\pi$ (kHz)	t (µs)	$\hat{\sigma}_\ell^\beta$	modes
2	2	1.201(4), 1.216(4)	-25	$2 \times (80 - 1080)$	$\hat{\sigma}_\ell^z$	ax-ip/lr-ip
3	3.5	0.1900(7), 1.608(6)	-20	$80 - 1280$	$\hat{\sigma}_\ell^y$	ax-ip/lr-ip
4	2	1.201(4), 1.216(4)	20/-20	$2 \times 2 \times (80 - 580)$	$\hat{S}_{\ell_1\ell_2}^z$	lr-ip/ur-ip
B.6(c,d) squeezing	2	1.201(4), 1.216(4)	25	530	$\hat{\sigma}_\ell^z$	lr-ip
B.6(c,d) tunnelling	2	1.201(4), 1.216(4)	-25	$2 \times (80 - 1080)$	$\hat{\sigma}_\ell^z$	lr-ip/ur-ip
B.8 squeezing	2	1.201(4), 1.216(4)	50	100	$\hat{\sigma}_\ell^z$	ax-ip
B.8 tunnelling	2	1.201(4), 1.216(4)	-25	$0 - 830$	$\hat{\sigma}_\ell^z$	ax-ip/lr-ip

Table II: Correlation coefficients between experimentally measured observables. We show the Pearson correlation coefficients r between gauge field observables ($\bar{s}_\ell^x/\bar{s}_{\ell_1\ell_2}^{xx}$) and average matter excitation $\bar{n}_{m_i}$.

Fig.	r_{ℓ,m_1}	r_{ℓ,m_2}	r_{m_1,m_2}
2	-0.94	0.92	-0.96
3	-0.78	0.78	-0.84
4(a)	0.88	-0.88	-0.87
4(b)	0.17	0.02	-0.19

Table III: Root mean squared error (rmse) between experimentally measured and numerically simulated values for observables ($\bar{s}_\ell^x/\bar{s}_{\ell_1\ell_2}^{xx}$, $\bar{n}_{m_1}$, and $\bar{n}_{m_2}$).

Fig.	rmse_ℓ	rmse_{m_1}	rmse_{m_2}
2	0.054	0.044	0.053
3	0.105	0.155	0.108
4(a)	0.106	0.139	0.107
