# Supplementary material for: Aharonov–Bohm interference in a ℤ2 lattice gauge theory on a hybrid qubit–oscillator quantum computer
Source: Nat Phys. 2026 Sep 25;22(10):1565–71. doi: 10.1038/s41567-026-03400-6 (PMC13658009; doi:10.1038/s41567-026-03400-6)
Supplement: Supplementary file 2 [file 41567_2026_3400_MOESM2_ESM.pdf]

nature portfolio

Peer Review File

Observation of Aharonov-Bohm Interference in a Z2 Lattice Gauge Theory on a Hybrid Qubit-Oscillator Quantum Computer

Corresponding Author: Dr Sebastian Saner

Version 0:

Reviewer comments:

Reviewer #1

(Remarks to the Author)
The manuscript by S. Saner et. al. reports an observation of Aharonov-Bohm interference in a Z2 lattice gauge theory (LGT) using a small-size trapped ion simulator, where gauge fields and matter fields are simulated by ion internal qubits and their external motional modes respectively. The qubit-oscillator hybrid system can be precisely coupled and controlled through lasers, allowing encoding for LGT and generating desired Hamiltonian. The manuscript is written well, and the results are clear and solid for supporting the main conclusions. I have a few concerns regarding the potential of this approach and the advantages over previous methods, and I do not find answers in the current manuscript but perhaps should be addressed before I give my recommendations.
1. There is a very recent work also using ions for simulating 2D LGT [Nature Physics 21, 570–576 (2025)], where qudits etc. are used to observe real-time dynamics associated with LGT. It's relevant but different in detailed implementation, however, showed similar capability to what have been shown in this manuscript. While this published work has not been mentioned in the current manuscript. But perhaps, it should be discussed. I am wondering what's the new thing which can be done based the approach introduced in this work compared to the qudit approach from the experimental perspective.
2. One nice feature from using harmonic oscillators is due to their high dimensions (infinite in theory), which are hard to be simulated classically. This work indeed employ this degrees of freedom, which is a good start. However, it did not show this advantage over using just qubits (or qudits encoded in internal states as in the paper mentioned above), where only |n=0> and |n=1> are used. In principle, higher fock states can be prepared and used, however, it seems that motional heating and/or dephasing already significantly impacts the experimental results even when only up to the n=1 state are used. As going to higher fock states, the performance is expected to be much worse. This raises a concern that if this approach can really go to somewhere which is difficult for classical computer to simulate.
3. In addition to 2, if many fock states are used in the future, how do the authors plan to characterize the dynamics of a qubit-oscillator system? In particular, is it required to read out an qubit-oscillator entangled state for LGT and how to do it efficiently?

Reviewer #2

(Remarks to the Author)
Please find my report in the attached PDF document.

Reviewer #3

(Remarks to the Author)
In this manuscript, Saner et al. consider a natural embedding of a Z2 lattice gauge theory in a quantum simulator comprised of trapped ions. In the particular approach taken here, each electric field variable (link) is encoded in the qubit state of one ion, and each matter field variable (lattice site) is encoded in a unique motional mode of the ion crystal. Two sets of experiments are carried out, one on a single qubit with two (active) motional modes and another with two qubits and two (active) motional modes. The authors show the ability to prepare various initial states and implement dynamics under a gauge invariant Hamiltonian describing the field-mediated hopping of matter fields.

On the one hand, the experiment could be viewed as a clever reformulation of straightforward dynamics of small (1 or 2 spins coupled to 2 bosonic modes) and simple quantum-optical systems in terms of lattice gauge theory. For example, the link-tunneling and Aharanov-Bohm experiments are both essentially characterizations of (detuned or resonant) Rabi oscillations of two-level systems (which are isolated within the larger quantum optical Hilbert space by symmetries, here

interpreted as gauge symmetries). With this in mind, I find it slightly hyperbolic to sell this paper as the first realization or (2+1)D lattice gauge theory, and therefore qualitatively beyond what has been done before in this regard. At the scale achieved here, there is not a particularly meaningful sense in which this paper explores (2+1)D physics [I realize the authors may object that the AB effect is intrinsically linked to being in (2+1)D, but I also think they will know what I mean]. Bearing this in mind, if this paper is to be interpreted as progress toward simulating (2+1)D lattice gauge theories, I think the validity of such an interpretation hinges on how credible it is to scale these methods up to many more qubits and modes, with adequate control over all of them to implement larger 2D-local geometries. I would personally like to see a more serious consideration of this issue in the main text. I would also like to see a clearer and earlier acknowledgement that these simulations involve at most two qubits and two modes (I had to read quite far before I realized this).

On the other hand, this paper is inarguably a beautiful demonstration of intricate control over a small trapped-ion quantum simulator, and a reasonable case could be made to publish in Nature Physics largely on those grounds. Therefore, I would be in favor of publication if the authors made some effort to address the concerns mentioned in the previous paragraph. The paper would be especially strengthened if the authors could draw connections between any specific AMO techniques pioneered here and the prospects for scalability into truly 2D systems.

Other specific comments on the paper:

In the paragraph starting “We consider an LGT that satisfies...”, I find the description a little confusing. In particular, it is stated that the gauge symmetry constrains the matter and link variables to be eigenstates of X and Q, respectively, but it’s really the (tensor product) Gauge operators that constrain the joint parity of these operators. In what sense is the gauge field “constrained to the two eigenstates of X” when the global state does not need to be an eigenstate of X? X just defines a convenient basis for writing down the (tensor-product) constraints.

Above Eq. 2, “The dynamics are restricted to the two states…” is confusing, the dynamics just produces the single time-dependent state that follows, which rabi oscillates between the two basis states contained therein (which are presumably what the above statement refers to).

The structure of the paper is a little bit disjointed, in that the section “Initializing and measuring the hybrid system” seems to refer specifically to initialization of the single-ion case, while the slightly different initialization in the 2-ion case is specifically called out separately in the section devoted to the Aharonov-Bohm effect.

Figure 3c, the authors mention the data does not agree with the expected values of both J and h, and say it likely implies h is not what’s expected. But isn’t h just a chosen detuning that’s known accurately? It might be nice to speculate why there is disagreement.

There are some clear discrepancies between data and theory in Fig. 4, do the authors have speculations about the dominant affects missed in the theory model?

Version 1:

Reviewer comments:

Reviewer #1

(Remarks to the Author)
I appreciate the detailed reply from the authors and they have properly addressed all my questions. The manuscript represents an outstanding demonstration that further extends the toolbox and applications of trapped ion systems. I am satisfied with the revisions and recommend publication.

Reviewer #2

(Remarks to the Author)
The authors addressed by comments to my fullest satisfaction, and I recommend the article for publication.

Reviewer #3

(Remarks to the Author)
I have read the responses of the Referee and modified manuscript. I find their description of both the scale of what's achieved---and more importantly how the demonstrated technical ingredients support a path to scaling---address my primary concern during initial review. All of my other more minor concerns have been addressed, and I believe the paper warrants publication in Nature Physics.

Open Access This Peer Review File is licensed under a Creative Commons Attribution 4.0 International License, which permits use, sharing, adaptation, distribution and reproduction in any medium or format, as long as you give appropriate credit to the original author(s) and the source, provide a link to the Creative Commons license, and indicate if changes were made.
In cases where reviewers are anonymous, credit should be given to 'Anonymous Referee' and the source.
The images or other third party material in this Peer Review File are included in the article's Creative Commons license, unless indicated otherwise in a credit line to the material. If material is not included in the article's Creative Commons license and your intended use is not permitted by statutory regulation or exceeds the permitted use, you will need to obtain permission directly from the copyright holder.
To view a copy of this license, visit https://creativecommons.org/licenses/by/4.0/

We thank all reviewers for their comments. We will reply to their remarks individually below (blue). Changes in the manuscript are marked in red.

Reviewer 1:

1. There is a very recent work also using ions for simulating 2D LGT [Nature Physics 21, 570–576 (2025)], where qudits etc. are used to observe real-time dynamics associated with LGT. It's relevant but different in detailed implementation, however, showed similar capability to what have been shown in this manuscript. While this published work has not been mentioned in the current manuscript. But perhaps, it should be discussed. I am wondering what's the new thing which can be done based the approach introduced in this work compared to the qudit approach from the experimental perspective.

We initially set aside this work, as the initial version of our manuscript focused on Z2 lattice gauge theories, whereas the paper primarily addresses U(1) lattice gauge theories, placing it within a broader body of work on trapped-ion and related platforms. Only later did we gain a different perspective, catalyzed by the context of the joint submissions and their shared emphasis on hybrid quantum systems for gauge theories, from which it is now clear that the section on real-time dynamics using Trotter expansions in qubit-qutrit systems is closely related to our work and therefore deserves appropriate credit in our manuscript.

We believe that the work of Meth et al., together with the simultaneous submission by Than et al. and our own manuscript, experimentally demonstrate the advantages of using hybrid quantum systems as quantum simulators while, at the same time, explore complementary hybrid platforms (i.e., qubit-oscillator versus qubit-qudit systems). As a result of this complementarity, different classes of lattice field theories can be simulated: qubit-qudit approaches targeting truncated representations of U(1) gauge theories (Meth et al.), and qubit-oscillator approaches targeting Yukawa couplings (Than et al.) or Z2 gauge theories with bosonic matter fields (our work).

From a technical perspective, there is an important distinction with respect to the work of Meth et al. in how the required gauge-matter coupling is generated. In our work, the gauge-invariant coupling between a qubit and two (a priori) infinite-dimensional oscillators (corresponding to a weight-3 Hamiltonian term), is implemented directly using an analog scheme based on parametric driving. This approach can readily incorporate a competing electric-field term without requiring Trotterization, as discussed in detail in our previous theoretical proposal [35]. In contrast, the work of Meth et al. requires decomposing this weight-3 term into a product of 24 two-qubit gates acting on selective subspaces of the qutrit, with further reductions to 6 two-qubit gates relying on the assumption that some terms can be neglected given perfect state preparation in a particular initial state. This Trotterization substantially increases the complexity when including also the remaining competing Hamiltonian terms. We think this might be the reason why only a single Trotter step could be realised experimentally, thus putting a stringent limit on the maximal time-evolution duration.

As demonstrated in our work, the real-time dynamics in our Z2 lattice gauge models are not limited by Trotter errors, nor by the accumulation of physical gate errors associated with decomposing each Trotter step. In fact, we use an analog approach

for the time-evolution and only utilise digital quantum gates in the preparation and measurement.

We have amended the manuscript and included the reference:

> Our approach is hybrid in two ways. First, it combines discrete and continuous variables to efficiently simulate the target LGT. Relatedly, qubit-qudit hybrid approaches have been explored, enabling complementary lattice gauge-theory studies of truncated U(1) models and real-time dynamics via Trotterised evolutions~\cite{meth2024simulating}. [...]

2. One nice feature from using harmonic oscillators is due to their high dimensions (infinite in theory), which are hard to be simulated classically. This work indeed employ this degrees of freedom, which is a good start. However, it did not show this advantage over using just qubits (or qudits encoded in internal states as in the paper mentioned above), where only |n=0> and |n=1> are used. In principle, higher fock states can be prepared and used, however, it seems that motional heating and/or dephasing already significantly impacts the experimental results even when only up to the n=1 state are used. As going to higher fock states, the performance is expected to be much worse. This raises a concern that if this approach can really go to somewhere which is difficult for classical computer to simulate.

While it is true that the main text of the manuscript does not present results for oscillators occupying Fock states beyond |n = 1>, as we decided to focus on the cleanest demonstration of gauge-invariant dynamics with the most transparent interpretation for general readers in terms of single Z2 charges, we have confirmed in the Method Section that the gauge-matter coupling induces the expected real-time dynamics also for n > 1.
This is now higlighted more explicitly in the section "Additonal Results And Future Work" section:

> Beyond the single-phonon matter excitations discussed so far, this approach also allows exploration of superpositions of Fock states with well-defined $\mathbb{Z}_2$ charges. In particular, we focus on squeezed matter states, which we investigate both experimentally and numerically, as detailed in Section VIII of the Methods.

In Sec. VIII of the Method Section, we go beyond this restriction by exploring the tunnelling of squeezed Z2 matter charges, which requires maintaining coherence over a linear superposition of, in principle, an infinite number of Fock states. As noted in the manuscript, the coupled dynamics of the gauge and matter fields is more sensitive to motional dephasing and heating. Nonetheless, some key features of the desired dynamics are already clearly observable.

We demonstrate that the gauge-invariant coupling leads to the expected Z2 charge dynamics, quantified by a periodic oscillation of the squeezing parameter between the two coupled oscillators. We further characterise the full Wigner function in phase space at selected evolution times, revealing the expected squeezing and the rotation of the squeezing axes induced by the gauge-invariant tunnelling. These observations are expected to generalise to other initial states in which the Z2 field is not projected onto the computational basis, but instead exhibits correlated dynamics constrained by Gauss law.

As also discussed in the Method Section, when the gauge field is initialised in the appropriate Gauss-law sector, its dynamics are consistent with the predicted effects of squeezing, leading to notable deviations from simple cosine-like oscillations for increasing squeezing parameters. For this observable, the impact of dephasing and heating becomes more pronounced, resulting in a reduction of the visibility. This limitation is attributable to the present experimental apparatus. By contrast, state-of-the-art trapped-ion platforms have demonstrated initial thermal state temperatures of nbar~0.02, heating rates as low as ~0.2 quanta/s (I. Pogorelov et al., PRX Quantum 2, 020343 (2021)) and significantly reduced dephasing (e.g. in Penning traps: V. Jarlaud et al., J. Phys. B: At. Mol. Opt. Phys. 54 015501 (2021)), which would enable a more quantitative benchmark of the effects of squeezing on the electric-field dynamics. Thus, there is a clear path towards future implementations without our limitations; our proof-of-principle experiment shows the utility of the applied techniques for this quantum simulation problem.

3. In addition to 2, if many Fock states are used in the future, how do the authors plan to characterize the dynamics of a qubit-oscillator system? In particular, is it required to read out an qubit-oscillator entangled state for LGT and how to do it efficiently?

State tomography of the qubits can, in principle, be performed straightforwardly. For a full tomographic characterisation of the oscillator, as shown in the Method Section, we employ a qubit coupled to the oscillator to reconstruct the characteristic function of the oscillator in phase space, which can then be related to the Wigner quasi-probability distribution via a Fourier transform.

Depending on the specific trapped-ion implementation, this readout qubit could be an auxiliary qubit. This would enable simultaneous measurements of both the gauge-field qubits and the matter-field oscillators at selected instants of the time evolution (future work), allowing correlations between matter and gauge fields to be directly extracted. In the present experiment, the qubit used for readout is the same as that in which the gauge field is previously encoded, which is thus collapsed and repurposed for measurement.

More generally, obtaining a full classical description of the quantum state via tomography becomes exponentially hard as the system size increases. The same growth of Hilbert space that enables quantum simulators to outperform classical methods also renders full state reconstruction intractable. This challenge arises in both purely qubit-based and hybrid systems. Importantly, however, we emphasise that a full tomographic characterisation is not required to observe the physical phenomena of interest. Instead, one can focus on specific gauge-invariant sectors, as is customary in lattice gauge theories.

For example, one may restrict attention to local gauge-invariant observables such as the local phonon number or the electric field and magnetic flux. To probe correlations, one could target Wilson lines or Wilson loops, which can be expressed as suitable correlation functions. Ultimately, a viable strategy is to establish trust in the simulator by performing full tomography on small-scale evolutions, and then to extract these physically relevant observables at larger scales.

Reviewer 2:

The spin-dependent force essentially realized by a scheme comparable to the Mølmer-Sørensen gate. Since coupling of the electronic states to the motional sidebands scales with $\sqrt{n}\,\eta$ with Lamb-Dicke parameter $\eta \ll 1$ and phonon number n, the effective Rabi *frequencies Ω1 and Ω2 are expected to change with the population of the mode. I believe the presented scheme will not be applicable when states |nmi⟩ with n>1 are involved. The authors should discuss potential strategies to mitigate this issue in future experiments, or provide alternative schemes to realize the desired interaction.*

The referee is correct in noting that the Rabi frequencies Ω1 and Ω2 depend on the Fock state populations. This leads to a modified scaling of the gauge-invariant tunnelling dynamics with the initial Fock state number (i.e., bosonic enhancement), which is in fact the desired and correct dynamical behaviour and not a limitation of the scheme. Higher Fock states can lead to richer gauge-invariant dynamics, as discussed in Ref. [35]. We emphasise that this dependence of the dynamics on n is built into the target lattice gauge model we aim to simulate, and does not imply any fundamental limitation for states with n > 1.
To draw a parallel following the analogy raised by the Referee, a similar situation occurs in the Mølmer–Sørensen scheme, which can mediate entanglement even when the phonons are in a thermal state. In our implementation, initial states with higher phonon occupation are acceptable as long as gauge symmetry is correctly imposed (see, for example, the discussion of squeezed Fock states in the Methods Section).

Regarding the validity of the effective gauge-invariant model and the role of the average phonon number, corrections that compromise gauge invariance arise from the breakdown of the Lamb–Dicke regime, $\eta^2 *(2n+1) \ll 1$. For our parameters, with $\eta \leq 0.05$, this condition remains reasonably well fulfilled even up to $n <= 25$. Again, following the above parallel, this limitation is analogous to the breakdown of the Mølmer-Sørensen scheme in extremely “hot” ion crystals. In this sense, the small value of η is advantageous.

Fig. 1(e) is a nice representation of the plaquette as a "loop" connecting two matter sites, however a connection with the 2D lattice shown in Fig. 1(a) would be appreciated. To which edges (vertices) are the two gauge fields (matter sites) assigned to?

In Fig. 1(a) we show a square plaquette consisting of four matter sites at the vertices of a 2D lattice, connected by four gauge-field links along the edges. Figure 1(e), by contrast, depicts a loop involving two matter sites connected by two gauge-field links, corresponding to the configuration realised in the present work. Both geometries form closed, gauge-invariant loops that can be pierced by a flux, thereby exhibiting quasi-two-dimensional properties.
However, we note that while the two-site loop in Fig. 1(e) captures the flux-sensitive paths, it does not correspond to a literal assignment of specific edges or vertices in Fig. 1(a) (although a mapping may exist in the limit of rapid tunnelling along one lattice direction). Figure 5 illustrates how a triangular loop, consisting of three matter sites and three gauge-field links, can be realized in practice, and the same construction principle directly extends to the square plaquette shown in Fig. 1(a).

The experiment is carried out on an apparatus featuring the macroscopic linear Paul trap described in the thesis of Thirumalai "High-fidelity mixed species entanglement of trapped ions", Oxford 2019. I am surprised by the heating rate of the axial center-of-mass (COM) motion with 300 quanta per second as stated by the authors. Why did the authors choose to use this mode at all, when the upper and lower radial modes perform equally well (see data in Fig. 4) and have comparable coherence times.

The experiments in Fig. 2 were carried out using one radial and the axial mode, as this was the configuration initially attempted (and we were historically used to using the COM mode). While the radial modes exhibit lower heating rates and comparable coherence times, the axial centre-of-mass (COM) mode benefits from a larger laser projection, leading to stronger coupling. For the experimental timescales relevant to Fig. 2, we found that the data from the axial mode was of sufficient quality, and we did not feel compelled to retake it on another pair of modes.

In the caption of Fig. 2 the authors mention a 12% correction applied in the displayed numerics. It is not clear to me which variable has been corrected (time axis / amplitude /decay / ...) and why is this correction justified.

The 12% correction mentioned in the caption of Fig. 2 corresponds to an adjustment of the tunnelling rate J in the numerics. Its microscopic origin is not fully understood at present, but it does not affect the interpretation of the targeted gauge physics; it merely accounts for a slight mismatch between the nominal simulation parameters and the observed experimental drive strength.

We have amended the caption of Fig. 2 to be more specific:

> ~~Solid lines show simulations using independently estimated parameters, except for the tunnelling rate \(J \), which is floated to match the experimental timescale. The required 12\% correction, applied in the displayed numerics, indicates a discrepancy relative to theoretical estimates and is left for future investigation.~~
>
> Solid lines show simulations using independently estimated parameters, except for the tunnelling rate \(J\), which is adjusted by 12\% to match the experimental timescale. This correction reflects a discrepancy between the expected drive strength and the measured dynamics and is left for future investigation.

One issue I see when simulating larger lattices and different geometries is mode crowding, and since coupling the electronic states with the motional states requires large intensities of the laser field, I expect the unwanted excitation of other modes and potentially large AC Stark shifts on the qubits encoding the link variables. Can the authors speculate on methods to tackle these issues?

This is a valuable insight. As we demonstrated previously, our parametric scheme scales linearly with eta (as discussed in the Supplement of [44]) and thus requires

modest beam powers in comparison to schemes that target dynamics scaling with eta^2 (e.g. quantum simulation of spin models). We agree that this does not solve all problems when aiming at effective models that use more common vibrational modes in larger crystals as oscillators of the lattice gauge theory. There are a few tricks one can play with regards to optimal compilation of intermediate-size problems. For instance, by optimising the mapping with the participation ratio of the ions to different modes in mind, one can partially alleviate the spectral mode crowding. Further, since the parametric coupling scheme is in essence derived from two Molmer-Sorensen type interactions; we believe that the mitigation techniques developed in the conventional entangling gate context could be modified and applied here too. For example, schemes that modulate amplitude or phase could be developed to suppress certain unwanted excitations (Steane et al. New J. Phys. 16, 053049 (2014), Blümel et al. npj Quantum Inf. 7, 147 (2021)). As for the AC stark-shifts the Referee mentions, these can be reduced by improving upon the single addressing quality, and the remainders need to be calibrated carefully in the experiment.

For larger sizes of the target model, the spectral mode crowding will likely be an ultimate roadblock. We have discussed in [35] an alternative scheme that requires microscopic control of the layout of trapping frequencies, and would most-likely require microfabricated segmented traps. In this case, one can always work with two common modes per pair of ions, and drive the parametric drivings selectively leading to the desired gauge-invariant dynamics.

We have incorporated this insight in the amended final paragraph of the main text.

I appreciate the resource estimates shown in Fig. 5, however the light green / mint colors make the numbers barely readable and should therefore be changed.

We have amended the outline color which should now also be visible in print.

The authors show the data of the experimentally measured expectation value ⟨¯sx l ⟩ in Fig. B.11 for the un-squeezed and squeezed matter with squeezing parameter ζ = 0.96 as a function of the tunneling duration; the theoretical calculations are shown in Fig. B.8. While the squeezed matter shows the predicted behavior (revival at Jt/π = 0.5 and Jt/π = 1.0), the oscillations for ζ = 0 warrant a more thorough discussion. Do the authors attribute their observation to finite temperature effects and motional heating? If so, why are they so pronounced when the thermal state is close to the ground state |0mi⟩.

As noted in the Supplemental Material, detailed simulations in the presence of squeezing are currently limited by our numerical library, and more quantitative studies of imperfections will require future updates or alternative approaches. In the ideal squeezed vacuum, the quantum state contains only even Fock states and thus zero Z2 charge, so gauge-invariant tunnelling is completely inhibited. In contrast, squeezing a thermal state or the presence of motional heating introduces small populations in odd Fock states, altering the Z2 charge and allowing finite tunnelling. While similar charge changes can occur when squeezing Fock state |1>, the residual population of even Fock states does not tunnel under gauge-invariant tunnelling, so the main signal is largely preserved. A quantitative confirmation of these effects will require further updates to our numerical machinery.

Reviewer 3:

In this manuscript, Saner et al. consider a natural embedding of a Z2 lattice gauge theory in a quantum simulator comprised of trapped ions. In the particular approach taken here, each electric field variable (link) is encoded in the qubit state of one ion, and each matter field variable (lattice site) is encoded in a unique motional mode of the ion crystal. Two sets of experiments are carried out, one on a single qubit with two (active) motional modes and another with two qubits and two (active) motional modes. The authors show the ability to prepare various initial states and implement dynamics under a gauge invariant Hamiltonian describing the field-mediated hopping of matter fields.
On the one hand, the experiment could be viewed as a clever reformulation of straightforward dynamics of small (1 or 2 spins coupled to 2 bosonic modes) and simple quantum-optical systems in terms of lattice gauge theory. For example, the link-tunneling and Aharanov-Bohm experiments are both essentially characterizations of (detuned or resonant) Rabi oscillations of two-level systems (which are isolated within the larger quantum optical Hilbert space by symmetries, here interpreted as gauge symmetries). With this in mind, I find it slightly hyperbolic to sell this paper as the first realization or (2+1)D lattice gauge theory, and therefore qualitatively beyond what has been done before in this regard. At the scale achieved here, there is not a particularly meaningful sense in which this paper explores (2+1)D physics [I realize the authors may object that the AB effect is intrinsically linked to being in (2+1)D, but I also think they will know what I mean]. Bearing this in mind, if this paper is to be interpreted as progress toward simulating (2+1)D lattice gauge theories, I think the validity of such an interpretation hinges on how credible it is to scale these methods up to many more qubits and modes, with adequate control over all of them to implement larger 2D-local geometries. I would personally like to see a more serious consideration of this issue in the main text. I would also like to see a clearer and earlier acknowledgement that these simulations involve at most two qubits and two modes (I had to read quite far before I realized this).

While the dynamics presented in the main text can be mapped onto an effective two-level system (|1,-,0> <-> |0, +, 1>) that forms part of the larger Hilbert space, this mapping relies on the preparation of specific initial states and on their subsequent time evolution being carried out with sufficiently low errors. The validity of such effective descriptions is therefore conditional, rather than generic. Characterising the experiments merely as Rabi oscillations would understate the complexity of the system and the combined role of gauge constraints, state-dependent mappings, and controlled spin-phonon dynamics that give rise to the oscillations of a charge attached to a string.

We acknowledge that the systems demonstrated in this work involve a hybrid system of at most two qubits and two-oscillator modes. We have thus amended the abstract and conclusion as follows to more clearly highlight the size of the utilised hybrid system early on.

> [...] probe dynamics obeying Gauss's law in a $\mathbb{Z}_2$ link and extend this to a loop geometry encoded in the hybrid system formed by two qubits and two oscillators, marking the first steps towards higher-dimensional LGTs.
>
> Utilising synthetic dimensions, we extended this to an elementary \(2+1\)D geometry, formed by two qubits and two harmonic oscillators on a two ion crystal, achieving the first quantum simulation of the real-time dynamics of a \(\mathbb{Z}_2\) loop.

Although we observe two-dimensional effects characteristic of magnetic fluxes and interference, the experiments correspond to a single Z2 loop. As such, the demonstrated dynamics could be interpreted either as those of a minimal two-dimensional loop/plaquette or equivalently as those of a one-dimensional system with periodic boundary conditions. In this sense, the concerns raised by the Referee regarding system size and dimensionality are reasonable.

Importantly, however, beyond these elementary demonstrations, we introduce and employ a broader set of experimental tools required to access, control, and characterise more complex hybrid qubit-oscillator setups in the future. These include the preparation of entangled gauge-field states in the magnetic-field basis, gauge-invariant parametric tunnelling, and phase-space-resolved measurements of the matter fields. As detailed in the Method Section, we develop techniques to characterise the real-time evolution of nontrivial oscillator states via Wigner-function tomography, enabling the study of dynamics involving higher phonon occupations and nonclassical states that go beyond simple two-level truncations. Together, these tools establish the experimental capability needed to explore genuinely interacting gauge-matter dynamics.

Finally, the question of scalability is addressed explicitly in the final paragraph of the main text (which we have substantially extended in this iteration as shown below) and in our earlier theoretical proposal [35]. Starting from the experimentally accessible ingredients demonstrated here, a straightforward scaling strategy consists of selectively resolving different qubit-oscillator subsystems thus mapping the one-dimensional ion chain geometry onto truly new synthetic dimensions. While ultimately limited by mode crowding, this approach should enable access to intermediate-size systems possibly leading to interesting manifestations of gauge dynamics that capture the essence of quasi-2d layouts. To overcome the ultimate limitations in system size due to mode crowding, we proposed in Ref. [35] a synthetic dimensional-reduction scheme in which collective vibrational modes are dimerised by locally engineering different trap frequencies, effectively grouping modes into independent matter sites. Each site can then be coupled to a pair of gauge qubits via state-dependent parametric tunnelling, ensuring local gauge invariance. This concept is further investigated in Ref. [51], where a period-4 dimerisation is proposed to enable the encoding of full loop chains. Although experimentally demanding, the essential ingredients required for these scaled implementations, namely parametric mode coupling, Bell-state flux encoding, gauge-invariant tunnelling, and flux-dependent interference, are all demonstrated in this work.

Further, we have amended the final paragraph of the main text to more clearly explain how the tools demonstrated in our work provide a path toward potential scaling and future approaches for larger lattice gauge systems.

~~Finally, synthetic dimensions open new possibilities for exploring LGTs in complex geometries. Engineering tunnelling between specific motional modes (representing matter sites) by addressing individual ions (representing links) enables various configurations, such as chains of connected loops~\cite{domanti2024dynamical}, triangular loops~\cite{homeier2021z2}, and even tetrahedral lattices, the simplest %three-dimensional instance of a $3+1$D LGT, all with modest experimental resources (Fig.~\ref{fig:extending_geometries}).~~

Finally, synthetic dimensions enable exploration of lattice gauge theories in complex geometries and provide a path to larger systems. The essential ingredients for this architecture: parametric mode coupling, Bell-state flux encoding, and preparation of non-trivial oscillator states are all demonstrated in the present work. Together with single-ion addressing, which is a standard capability in trapped-ion platforms, these ingredients provide a straightforward scaling strategy based on selectively resolving qubit-oscillator subsystems in a long ion chain, effectively mapping a one-dimensional ion chain onto new synthetic dimensions and geometries.
By engineering tunnelling between selected motional modes (matter sites) and selected ions (links), one can then realise chains of connected loops~\cite{domanti2024dynamical}, triangular loops~\cite{homeier2021z2}, and tetrahedral lattices, the simplest three-dimensional $3+1$D LGT, all with modest experimental resources (Fig.~\ref{fig:extending_geometries}). While ultimately limited by mode crowding, this approach allows access to moderately large systems. To extend further, Refs.~\cite{bazavan2022synthesizing, domanti2024dynamical} propose synthetic dimensional reduction via dimerised vibrational modes with locally engineered trap frequencies, grouping modes into independent matter sites. Each site is then coupled to a pair of gauge qubits via state-dependent parametric tunnelling to ensure local gauge invariance.

We have outlined what we believe to be a promising path toward larger and higher-dimensional lattice geometries, though the ultimate feasibility will of course depend on future theoretical investigation and, more importantly, on further experimental developments.

On the other hand, this paper is inarguably a beautiful demonstration of intricate control over a small trapped-ion quantum simulator, and a reasonable case could be made to publish in Nature Physics largely on those grounds. Therefore, I would be in favor of publication if the authors made some effort to address the concerns mentioned in the previous paragraph. The paper would be especially strengthened if the authors could draw connections between any specific AMO techniques pioneered here and the prospects for scalability into truly 2D systems.

We appreciate the reviewers' assessment. We have tried to strengthen the text accordingly and explain/draw the connections more precisely as discussed in the previous paragraph.

Other specific comments on the paper:

In the paragraph starting "We consider an LGT that satisfies...", I find the description a little confusing. In particular, it is stated that the gauge symmetry constrains the

matter and link variables to be eigenstates of X and Q, respectively, but it's really the (tensor product) Gauge operators that constrain the joint parity of these operators. In what sense is the gauge field "constrained to the two eigenstates of X" when the global state does not need to be an eigenstate of X? X just defines a convenient basis for writing down the (tensor-product) constraints.

We agree that the way we introduced this in the text targeting non-experts is indeed incorrect. We have clarified the text as follows:

> ~~We consider an LGT that satisfies a local $\mathbb{Z}_2$ gauge symmetry, which in the single-link case is generated by $\hat{G}_{m_i} = \hat{\sigma}^x_\ell \hat{P}_{m_i}$ at each site and at all times, where $\hat{P}_{m_i} = \exp({\rm i} \pi \hat{a}_{m_i}^\dagger \hat{a}_{m_i}^{})$ is the matter parity. Physical states obey Gauss's law and are therefore eigenstates of $\hat{G}_{m_i}$, constraining the gauge field to the two eigenstates of $\hat{\sigma}_{\ell}^x$; $ \ket{+_{\ell}}$ and $ \ket{-_{\ell}}$ corresponding to the presence ($+$) or absence ($-$) of an electric-field line connecting the two matter sites. The dynamical charges are likewise constrained to eigenstates of $\hat{P}_{m_i} = 1 - 2 \hat{Q}_{m_i}$. Here, \(\hat{Q}_{m_i} \) is the bosonic \(\mathbb{Z}_2\) charge operator at site \(m_i \), with eigenvalues \(q_{m_i} \in \{0,1\} \). States with an even (odd) number of bosons $\ket{2k_{m_i}}(\ket{(2k+1)_{m_i}})$ have charge \(q_{m_i} = 0 \) (\(1\)).~~
>
> We consider an LGT that satisfies a local $\mathbb{Z}_2$ gauge symmetry, which in the single-link case is generated by $\hat{G}_{m_i} = \hat{\sigma}^x_\ell \hat{P}_{m_i}$ at each site and at all times, where $\hat{P}_{m_i} = \exp\!\left(i\pi \hat{a}_{m_i}^\dagger \hat{a}_{m_i} \right) = 1 - 2 \hat{Q}_{m_i}$ is the matter parity. In this representation, $\hat{\sigma}^x_\ell$ provides a convenient basis for the gauge field, with eigenstates $\ket{+_\ell}$ and $\ket{-_\ell}$, corresponding to the presence $(+)$ or absence $(-)$ of an electric-field line connecting the two matter sites. Similarly, $\hat{Q}_{m_i}$ defines a convenient local basis for the matter (charge) degrees of freedom, with eigenvalues $q_{m_i} \in \{0,1\}$, such that states with an even (odd) number of bosons $\ket{2k_{m_i}}$ ($\ket{(2k+1)_{m_i}}$) have charge $q_{m_i} = 0$ (1). However, while physical states obey Gauss's law and must therefore be eigenstates of $\hat{G}_{m_i}$, the gauge and matter field can be entangled and need not be eigenstates of $\hat{\sigma}^x_\ell$ or $\hat{P}_{m_i}$ individually.

Above Eq. 2, "The dynamics are restricted to the two states…" is confusing, the dynamics just produces the single time-dependent state that follows, which rabi oscillates between the two basis states contained therein (which are presumably what the above statement refers to).

We have rewritten this sentence to avoid any possible confusion. What we meant is that the dynamics of a particular initial state takes place in a two-level subspace.

> The dynamics are restricted to the two-level subspace [...]

The structure of the paper is a little bit disjointed, in that the section "Initializing and measuring the hybrid system" seems to refer specifically to initialization of the single-ion case, while the slightly different initialization in the 2-ion case is specifically called out separately in the section devoted to the Aharonov-Bohm effect.

We understand the Referee's concern, although we have not found a better way to present things without this small inconsistency. Advancing the preparation of for the Z2 loop before the physics is discussed will likely become very challenging for a potential reader who is not an expert. We amended the section title to:

Initialising and measuring the single link hybrid system

Figure 3c, the authors mention the data does not agree with the expected values of both J and h, and say it likely implies h is not what's expected. But isn't h just a chosen detuning that's known accurately? It might be nice to speculate why there is disagreement.

The phrasing in the previous version of the manuscript was slightly imprecise: While the microscopic detuning h is precisely known, deviations from the ideal Z2 dynamics arise from imperfect initial state preparation, neglected terms in the parametric scheme, and additional sources of noise, such as heating. These effects are explicitly included in our numerical simulations, which reproduce the experimental data using the same h as experimentally set and yield Ω_0 values in good agreement with the measurements. From a Z2 LGT perspective, these corrections can then be interpreted as an effective reduction of the electric-field strength h_eff in the realisation of the quantum simulation. We amend the last sentence of the caption as follows:

~~We do, however, find a deviation from the analytic theory $\Omega_0/J = \sqrt{1+(h/J)^2}$ (dotted line), which indicates that the the effective electric field term that is introduced has a smaller magnitude than anticipated from the analytic expression.~~

Within the $\mathbb{Z}_2$ lattice gauge theory framework, where Ω_0 is expected to follow the analytic prediction $\Omega_0 = J \sqrt{1+(h/J)^2}$, the observed corrections can be interpreted as a reduction of the electric field, $h_{\mathrm{eff}} < h$, in the realised quantum simulation.

There are some clear discrepancies between data and theory in Fig. 4, do the authors have speculations about the dominant affects missed in the theory model?

We believe that the discrepancies in the Φ flux states (left column) indicates that our assumptions about the magnitude or structure of the dephasing noise present in our hybrid system were too simplistic, leading us to underestimate the decay of the tunnelling oscillation amplitude.

We suspect that the discrepancies in the Ψ flux state (right column) arises from several effects during the state preparation and readout steps, each contributing to a similar degree and leading to the offset observed. In particular, preparing the state $\ket{1,\downarrow,\downarrow,0}$ requires a lengthy sequence that temporarily passes through $\ket{1,\downarrow,\uparrow,0}$, as discussed in the Methods section. We may be overestimating the fidelity with which this sequence is executed. Moreover, the additional time required during both the preparation and readout steps can lead to excess motional heating, contributing to incoherent noise in the system.

NPHYS-2025-09-03958 Real-Time Observation of Aharonov-Bohm Interference in a Z2 Lattice Gauge Theory on a Hybrid Qubit-Oscillator Quantum Computer

In this manuscript the authors present the quantum simulation of a $\mathbb{Z}_2$ lattice gauge theory on a trapped-ion platform. The gauge field(s) / link variable(s) are encoded in the electronic states of trapped $^{88}Sr^+$ ions, while occupation of the matter sites is encoded in the motional modes, making their platform a hybrid qubit-qudit quantum processor. The motion is excited using a quad-chromatic laser field, thereby introducing a spin-dependent force on the ion(s). First, the authors demonstrate the dynamic tunneling of matter between two sites connected by a single $\mathbb{Z}_2$ link for a) vanishing and b) non-vanishing electric field energy. Second, Aharonov-Bohm interference is observed in the time dynamics of the matter field in a $\mathbb{Z}_2$ loop. This experiment demonstrates the interplay of charge and flux – a genuine 2D effect – on a trapped-ion quantum simulator.

The simulation of the bosonic matter is restricted to the subspace $|0m_i\rangle\,/\,|1m_i\rangle$, effectively restricting the matter on each site to a single boson, which could also be encoded in the electronic states of another ion. I believe the full simulation could be carried out on a register of three ions (matter and link), or possibly four ions for the $\mathbb{Z}_2$ loop. Expanding the simulation beyond the subspace $|0m_i\rangle\,/\,|1m_i\rangle$ would significantly strengthen the manuscript.

The authors provide an extensive methods section, along with additional materials and measurement data in the supplementary material (SM). The results presented there are impressive and should be incorporated into the main text, or at least more prominently highlighted to encourage readers to explore the SM further.

I recommend the article for publication with a few minor revisions, which I hope the authors will address before their final submission. Their work is both timely and novel, and is of interest to the readers of Nature Physics.

> The spin-dependent force essentially realized by a scheme comparable to the Mølmer-Sørensen gate. Since coupling of the electronic states to the motional sidebands scales with $\sqrt{\eta}n$ with Lamb-Dicke parameter $\eta \ll 1$ and phonon number n, the effective Rabi frequencies Ω_1 and Ω_2 are expected to change with the population of the mode. I believe the presented scheme will not be applicable when states $|nm_i\rangle$ with $n > 1$ are involved. The authors should discuss potential strategies to mitigate this issue in future experiments, or provide alternative schemes to realize the desired interaction.

> Fig. 1(e) is a nice representation of the plaquette as a "loop" connecting two matter sites, however a connection with the 2D lattice shown in Fig. 1(a) would be appreciated. To which edges (vertices) are the two gauge fields (matter sites) assigned to?

> The experiment is carried out on an apparatus featuring the macroscopic linear Paul trap described in the thesis of Thirumalai *"High-fidelity mixed species entanglement of trapped ions"*, Oxford 2019. I am surprised by the heating rate of the axial center-of-mass (COM) motion with 300 quanta per second as stated by the authors. Why did the authors choose to use this mode at all, when the upper and lower radial modes perform equally well (see data in Fig. 4) and have comparable coherence times?

> In the caption of Fig. 2 the authors mention a *12% correction applied in the displayed numerics*. It is not clear to me which variable has been corrected (time axis / amplitude / decay / ...) and why is this correction justified.

> One issue I see when simulating larger lattices and different geometries is mode crowding, and since coupling the electronic states with the motional states requires large intensities of the laser field, I expect the unwanted excitation of other modes and potentially large AC Stark shifts on the qubits encoding the link variables. Can the authors speculate on methods to tackle these issues?

> I appreciate the resource estimates shown in Fig. 5, however the light green / mint colors make the numbers barely readable and should therefore be changed.

> The authors show the data of the experimentally measured expectation value $\langle \bar{s}_l^x \rangle$ in Fig. B.11 for the un-squeezed and squeezed matter with squeezing parameter $\zeta = 0.96$ as a function of the tunneling duration; the theoretical calculations are shown in Fig. B.8. While the squeezed matter shows the predicted behavior (revival at $Jt/\pi = 0.5$ and $Jt/\pi = 1.0$), the oscillations for $\zeta = 0$ warrant a more thorough discussion. Do the authors attribute their observation to finite temperature effects and motional heating? If so, why are they so pronounced when the thermal state is close to the ground state $|0m_i\rangle$?
